# Cesarean Section and Rate of Subsequent Stillbirth, Miscarriage, and Ectopic Pregnancy: A Danish Register-Based Cohort Study

**DOI:** 10.1371/journal.pmed.1001670

**Published:** 2014-07-01

**Authors:** Sinéad M. O'Neill, Esben Agerbo, Louise C. Kenny, Tine B. Henriksen, Patricia M. Kearney, Richard A. Greene, Preben Bo Mortensen, Ali S. Khashan

**Affiliations:** 1 National Perinatal Epidemiology Centre, Anu Research Centre, Department of Obstetrics and Gynaecology, Cork University Maternity Hospital, Cork, Ireland; 2 National Centre for Register-based Research, Aarhus University, Aarhus, Denmark; 3 Centre for Integrated Register-based Research, Aarhus University, Aarhus, Denmark; 4 Irish Centre for Fetal and Neonatal Translational Research, University College Cork, Cork, Ireland; 5 Perinatal Epidemiology Research Unit, Department of Paediatrics, Aarhus University Hospital, Skejby, Denmark; 6 Department of Epidemiology and Public Health, University College Cork, Cork, Ireland; University of Queensland, Australia

## Abstract

**Background:**

With cesarean section rates increasing worldwide, clarity regarding negative effects is essential. This study aimed to investigate the rate of subsequent stillbirth, miscarriage, and ectopic pregnancy following primary cesarean section, controlling for confounding by indication.

**Methods and Findings:**

We performed a population-based cohort study using Danish national registry data linking various registers. The cohort included primiparous women with a live birth between January 1, 1982, and December 31, 2010 (*n* = 832,996), with follow-up until the next event (stillbirth, miscarriage, or ectopic pregnancy) or censoring by live birth, death, emigration, or study end. Cox regression models for all types of cesarean sections, sub-group analyses by type of cesarean, and competing risks analyses for the causes of stillbirth were performed. An increased rate of stillbirth (hazard ratio [HR] 1.14, 95% CI 1.01, 1.28) was found in women with primary cesarean section compared to spontaneous vaginal delivery, giving a theoretical absolute risk increase (ARI) of 0.03% for stillbirth, and a number needed to harm (NNH) of 3,333 women. Analyses by type of cesarean section showed similarly increased rates for emergency (HR 1.15, 95% CI 1.01, 1.31) and elective cesarean (HR 1.11, 95% CI 0.91, 1.35), although not statistically significant in the latter case. An increased rate of ectopic pregnancy was found among women with primary cesarean overall (HR 1.09, 95% CI 1.04, 1.15) and by type (emergency cesarean, HR 1.09, 95% CI 1.03, 1.15, and elective cesarean, HR 1.12, 95% CI 1.03, 1.21), yielding an ARI of 0.1% and a NNH of 1,000 women for ectopic pregnancy. No increased rate of miscarriage was found among women with primary cesarean, with maternally requested cesarean section associated with a decreased rate of miscarriage (HR 0.72, 95% CI 0.60, 0.85). Limitations include incomplete data on maternal body mass index, maternal smoking, fertility treatment, causes of stillbirth, and maternally requested cesarean section, as well as lack of data on antepartum/intrapartum stillbirth and gestational age for stillbirth and miscarriage.

**Conclusions:**

This study found that cesarean section is associated with a small increased rate of subsequent stillbirth and ectopic pregnancy. Underlying medical conditions, however, and confounding by indication for the primary cesarean delivery account for at least part of this increased rate. These findings will assist women and health-care providers to reach more informed decisions regarding mode of delivery.

*Please see later in the article for the Editors' Summary*

## Abstract 2

Louise Kenny and colleagues conduct a population-based cohort study in Denmark to assess the likelihood of stillbirth, miscarriage, and ectopic pregnancy following cesarean section compared to women who gave birth by vaginal delivery.

*Please see later in the article for the Editors' Summary*

## Editors' Summary

**Background:**

Globally, increasing numbers of babies are being delivered by cesarean section (a surgical operation in which the baby is delivered through a cut made in the mother's abdomen and womb) instead of naturally through their mother's vagina. In England in 2010, for example, nearly 25% of all babies were delivered by cesarean section (also called C-section) compared to only 2% in the 1950s; in China and some parts of South America cesarean rates are now between 40% and 50%. A cesarean section is usually performed when a vaginal birth would endanger the life of the mother or her unborn child because, for example, the baby is in the wrong position. Some cesareans are performed as emergency procedures, but others are planned in advance when the need for the operation becomes clear during pregnancy (an elective cesarean). Some planned cesarean sections are also undertaken because the mother has requested a cesarean delivery in the absence of any medical reasons for such a delivery.

**Why Was This Study Done?:**

Cesarean sections save lives but do they have any negative impacts on the outcome of subsequent pregnancies? With so many cesarean sections being undertaken, it is important to be sure that the procedure does not increase the rates of subsequent miscarriage, stillbirth, or ectopic pregnancy. Miscarriage—the loss of a fetus (developing baby) that is unable to survive independently—is the commonest complication of early pregnancy, affecting about one in five women who know they are pregnant. Stillbirth is fetal death after about 20–24 weeks of pregnancy; the exact definition of stillbirth varies between countries. About four million stillbirths occur each year worldwide. Ectopic pregnancy—development of the fetus outside the womb—occurs in 1%–2% of all pregnancies. In this population-based cohort study, the researchers investigate the rates of subsequent stillbirth, miscarriage, and ectopic pregnancy following a cesarean section among women living in Denmark. A population-based cohort study determines the baseline characteristics of the individuals in a population, and then follows the population over time to see whether specific characteristics are associated with specific outcomes.

**What Did the Researchers Do and Find?:**

The researchers obtained data for 832,996 women from Danish national registers about their first live birth (including whether they had a cesarean) then followed the women (again using the registers) until they had a stillbirth, miscarriage, or ectopic pregnancy, or a second live birth. The researchers used these data and statistical models to estimate the risk of stillbirth, miscarriage, and ectopic pregnancy following a cesarean compared to a spontaneous vaginal delivery after controlling for the possibility that the cesarean was performed because of an indication that might increase the risk of a subsequent event (confounding). Women who had had a cesarean had a 14% increased risk of a stillbirth in their next pregnancy compared to women who had had a vaginal delivery, corresponding to an absolute risk increase of 0.03%. In other words, 3,333 women would need to have a cesarean to result in one extra stillbirth in subsequent pregnancy (a “number needed to harm” of 3,333). Compared to vaginal delivery, having a cesarean increased the risk of a subsequent ectopic pregnancy by 9% (an absolute risk increase of 0.1% and a number needed to harm of 1,000) but did not increase the rate of subsequent miscarriages.

**What Do These Findings Mean?:**

These findings show that, among women living in Denmark, cesarean section is associated with a slightly increased rate of subsequent stillbirth and ectopic pregnancy. Part of this increase can be accounted for by underlying medical conditions and by confounding by the indication for the primary cesarean section. The accuracy of these findings may be affected by limitations in the study such as incomplete data on some factors (for example, the smoking history of the mother) that might have affected the risk of stillbirth, miscarriage, and ectopic pregnancy, and by misclassification or underreporting of the study outcomes. Given the global increase in cesarean rates, these findings suggest that cesarean delivery is not associated with an increased rate of subsequent stillbirth, miscarriage, or ectopic pregnancy, an important finding for both expectant mothers and health-care professionals that nonetheless needs to be confirmed in further large-scale studies. Finally, these findings highlight the need for women to consider all their options thoroughly before requesting a cesarean section on non-medical grounds.

**Additional Information:**

Please access these websites via the online version of this summary at http://dx.doi.org/10.1371/journal.pmed.1001670.

- The American Congress of Obstetricians and Gynecologists provides patient fact sheets on cesarean birth, miscarriage, and ectopic pregnancy
- The US-based non-profit Nemours Foundation provides information about cesarean sections, miscarriage and stillbirth, and ectopic pregnancy (in English and Spanish)
- The UK National Health Service Choices website provides information for patients about cesarean section, miscarriage, stillbirth, and ectopic pregnancy
- MedlinePlus provides links to additional resources about cesarean section, miscarriage, stillbirth, and ectopic pregnancy (in English and Spanish)
- The UK non-profit organization Healthtalkonline provides personal stories about cesarean delivery, miscarriage, and stillbirth

## Introduction

The overall rates of cesarean delivery are increasing significantly in many parts of the world [1]. In England in 2010, the proportion of total births by cesarean section was almost 25%, compared with just 2% in the 1950s [2]. In the United States and Australia rates of greater than 33% have been reported [3],[4], and in China [5] and parts of South America, including Brazil and Paraguay [6], cesarean rates of between 40% and 50% are common. Concerns have been expressed regarding the impact of a cesarean section on subsequent pregnancy outcome [1],[7]–[9], particularly the rate of subsequent stillbirth, miscarriage, and ectopic pregnancy. Hypothesized biological mechanisms include placental abnormalities, prior infection, and adhesion formation due to cesarean section [10]–[12]. Stillbirth is one of the most devastating adverse pregnancy outcomes, with over 4 million occurring each year worldwide [13]. Miscarriage is the most common complication of pregnancy in the first trimester, with most studies reporting that one in five clinical pregnancies will end in miscarriage [14]–[16], but rates of one-third have been cited in prospective studies on early pregnancy loss [17]. Ectopic pregnancy is one of the leading causes of maternal morbidity and mortality, occurring in 1%–2% of all pregnancies [18]. Therefore, any potential association between cesarean section and subsequent stillbirth, miscarriage, or ectopic pregnancy is of significant concern.

One of the largest studies to date using high-quality maternity registry data found an increased rate of stillbirth following cesarean section in a cohort of more than 100,000 women (hazard ratio [HR] 2.23, 95% CI 1.48, 3.36) [19],[20]. Findings from a recent systematic review and meta-analysis [21] reported a 23% increased odds of stillbirth following cesarean section (pooled odds ratio [OR] 1.23, 95% CI 1.08, 1.40). Similarly Flenady et al. [12] reported a 21% increased odds of stillbirth (pooled OR 1.21, 95% CI 1.07, 1.37) in their meta-analysis. Both reviews, however, were limited by significant heterogeneity between the included studies, such as variations in the cause and timing of stillbirth (of which there are over 35 classification systems in use) [22]. Furthermore, studies investigating the rate of miscarriage were restricted by methodological limitations including small sample size, lack of detailed obstetric data (including the indication for cesarean section), and the inability to adjust for key potential confounders such as a history of pregnancy loss. A recent meta-analysis examining the likelihood of ectopic pregnancy following cesarean section [23] found no increased odds (pooled OR 1.05, 95% CI 0.51, 2.15); however, the results were limited by the quality of epidemiological studies to date, with fewer than half adjusting for potential confounders.

Confounding by indication may occur when a cesarean section is performed as a result of a clinical characteristic or medical condition being present that “indicates” the need for a cesarean section and, at the same time, increases the risk of the outcome under study [24]. A recent review recommended further research into the association between cesarean section and risk of subsequent adverse pregnancy outcome, with an emphasis on examining the indication for cesarean section and whether or not this is a confounding factor [25]. Therefore, we conducted the largest population-based cohort study to date, to our knowledge, using nationwide registry data including women with a primary cesarean section to assess the likelihood of stillbirth, miscarriage, and ectopic pregnancy compared to women with a primary vaginal delivery.

## Methods

### Study Design and Data Sources

A population register-based cohort study using Danish Civil Registration System (CRS) data [26] was conducted and linked to registers including the Danish National Hospital Register (NHR) [27],[28], the Danish Medical Birth Registry (MBR) [29],[30], and the Danish Register of Causes of Death [31],[32], and Statistics Denmark [33]. The CRS was established in 1968 and allows for the long-term follow-up of individuals and accurate linkage between and within registers through the use of a unique personal identification number known as the civil personal registration (CPR) number, which is assigned to all individuals alive and resident in Denmark [26]. Other information contained in the CRS includes name, gender, date of birth, place of birth, place of residence, vital status (updated continuously), CPR numbers of parents and spouses, along with more than 150 additional variables [34]. The Danish registry data, in particular miscarriage reporting data, have been validated for use in epidemiological research [30],[35],[36].

### Study Population and Follow-Up

**Figure 1:**
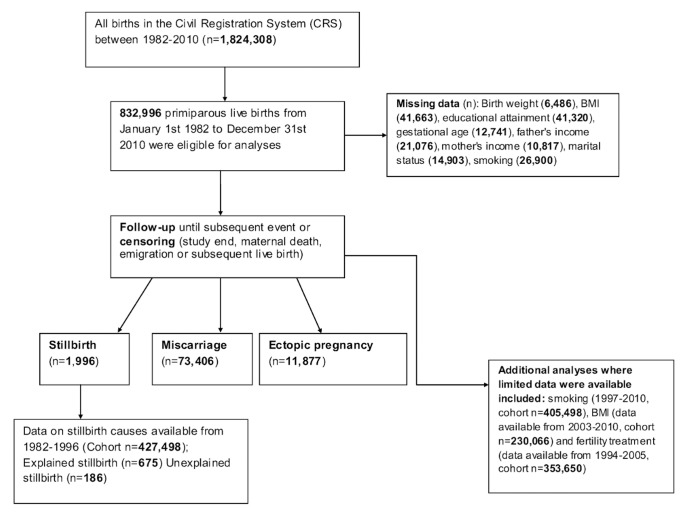
Flow diagram of study participants.

We identified a cohort of all live births to primiparous women in Denmark between January 1, 1982, and December 31, 2010, using the CRS and linked registers (Figure 1). Index live births included singleton and multiple gestation (twins or more) deliveries. The study population for analyses consisted of 832,996 women who were followed up until subsequent stillbirth, miscarriage, or ectopic pregnancy or until censoring by live birth, maternal death, emigration, or study end (December 31, 2010). Termination of pregnancy (induced abortion) is legal in Denmark; however, for the current analysis we did not have access to such data. Therefore, in our primary analysis, if there were any women who opted to have a termination of pregnancy, these women would still have been followed-up until the pregnancy event of interest (stillbirth, miscarriage, or ectopic pregnancy) or study end.

### Mode of Delivery

Women were categorized according to mode of delivery in the first live birth: spontaneous vaginal delivery (SVD) (reference group), operative vaginal delivery (vacuum- or forceps-assisted vaginal delivery), emergency cesarean section, elective cesarean section (pre-planned, generally as a result of one or more specific medical indications), and maternally requested cesarean section (data for maternally requested cesarean section were available from 2002–2010 only). It must be stated that the variable for maternally requested cesarean is recorded as a tick-box option and therefore may be a mix of both the clinician's opinion and maternal request.

### Outcome Classification

Diagnostic information is based on the Danish version of the International Classification of Diseases (ICD) 8th revision (from 1977 to 1993), and the ICD 10th revision (from 1994 to the present time).

Stillbirth was defined as the death of a fetus at 28 wk gestation or later in Denmark until 2004 [37]. After this period, the National Board of Health changed the definition to the death of a fetus born after 22 completed weeks' gestation. Stillbirth was recorded according to ICD-8 code 779 and ICD-10 code P95. Stillbirth was further categorized into “explained” (antenatal complications, complications of delivery, congenital malformations of the fetus, maternal illness, or injury to the mother) or “unexplained” (unknown causes of death, cancers [including malignant neoplasm of the bone or spinal cord] or other benign neoplasms of unspecified organs or tissues, post-maturity, and haemorrhages, including co-twin, subarachnoid, other intracranial, newborn, or unspecified haemorrhages) according to the methods outlined by King-Hele et al. [38]. Data for all stillbirths were available from 1982–2010; however, data on the specific causes of stillbirth were available from 1982–1996 only. Data from 1997–2010 were not available for the current analyses.

Miscarriage was defined as a pregnancy spontaneously ended before 28 wk gestation until April 2004, and before 22 wk gestation from 2004 onwards in Denmark [35]. Miscarriage was recorded according to the ICD-8 codes 634.2 and 643 (643.0, 643.1, 643.2, and 643.9) and ICD-10 codes O02 and O03.

Ectopic pregnancy was recorded using the ICD-8 code 631 and ICD-10 code O00.

### Covariate Definitions

Maternal covariates included maternal age, maternal country of origin, birth type (singleton or multiple delivery), smoking status (data from 1997–2010), maternal body mass index (BMI) (data from 2003–2010), obstetric history, history of fertility treatment (data from 1994–2005), maternal educational attainment, mother's and father's gross income, mother's marital status, and co-morbidities in the first live birth. Infant covariates included birth weight, length, sex, and gestational age (Table 1).

**Table 1:** Baseline characteristics of the cohort for the first live birth.

Characteristic	Spontaneous Vaginal Delivery	Operative Vaginal Delivery	Emergency Cesarean Section	Elective Cesarean Section	Maternally Requested Cesarean Section*
**Total**	607,252 (72.90)	79,827 (9.58)	104,091 (12.50)	38,950 (4.68)	2,876 (1.11)
**Maternal age, years**					
<20	21,883 (85.00)	1,255 (4.87)	1,994 (7.75)	585 (2.27)	28 (0.52)
20–25	210,595 (80.08)	17,828 (6.78)	25,840 (9.83)	8,356 (3.18)	364 (0.68)
26–30	245,579 (72.51)	35,687 (10.54)	41,448 (12.24)	15,134 (4.47)	812 (0.74)
31–35	100,283 (64.74)	18,995 (12.26)	24,760 (15.98)	9,888 (6.38)	977 (1.46)
36–40	25,427 (57.79)	5,326 (12.10)	8,574 (19.49)	4,130 (9.39)	545 (2.71)
41+	3,485 (51.99)	736 (10.98)	1,475 (22.01)	857 (12.79)	150 (4.88)
**Maternal origin**					
Denmark	527,328 (72.78)	79,102 (9.68)	90,540 (12.50)	34,340 (4.74)	2,249 (1.04)
Other	78,003 (80.54)	525 (0.54)	13,243 (13.67)	4,470 (4.62)	615 (0.63)
Unknown	1,921 (74.43)	200 (7.75)	308 (11.93)	140 (5.42)	12 (1.61)
**Birth type**					
Singleton	601,111 (73.51)	79,455 (9.72)	98,972 (12.10)	35,462 (4.34)	2,700 (1.07)
Multiple (twins or more)	6,141 (40.15)	372 (2.43)	5,119 (33.47)	3,488 (22.80)	176 (2.74)
**Maternal smoking** a					
Yes	40,919 (68.51)	6,971 (11.67)	8,513 (14.25)	2,966 (4.97)	362 (1.13)
No	211,833 (66.43)	41,639 (13.06)	46,472 (14.57)	16,580 (5.20)	2,343 (1.11)
Unknown	17,722 (65.88)	2,849 (10.59)	4,509 (16.76)	1,649 (6.13)	171 (1.10)
**Maternal BMI** b **(kg/m^2^)**					
<18.5	9,673 (68.49)	1,874 (13.27)	1,609 (11.39)	758 (5.37)	209 (1.48)
18.5–25	84,390 (65.12)	17,379 (13.41)	19,072 (14.72)	7,092 (5.47)	1,667 (1.29)
26–30	17,482 (59.93)	3,757 (12.88)	5,812 (19.92)	1,707 (5.85)	414 (1.42)
31–35	6,011 (57.59)	1,199 (11.49)	2,409 (23.08)	662 (6.34)	157 (1.50)
36–40	1,877 (54.15)	360 (10.39)	930 (26.83)	250 (7.21)	49 (1.41)
41+	827 (51.56)	153 (9.54)	472 (29.43)	128 (7.98)	24 (1.50)
Unknown	27,950 (67.09)	4,900 (11.76)	6,218 (14.92)	2,297 (5.51)	298 (0.72)
**Obstetric history** c					
Previous stillbirth	2,354 (58.38)	130 (3.22)	650 (16.12)	826 (20.49)	72 (6.09)
Previous miscarriage	4,549 (64.36)	783 (11.08)	1,180 (16.69)	521 (7.37)	35 (1.51)
Previous ectopic pregnancy	9,797 (65.76)	1,588 (10.66)	2,361 (15.85)	1,066 (7.16)	86 (1.75)
**Mother's educational attainment**					
Primary education	164,565 (77.21)	14,899 (6.99)	24,502 (11.50)	8,679 (4.07)	501 (1.14)
High school	278,573 (72.00)	39,660 (10.25)	49,058 (12.68)	18,423 (4.76)	1,178 (1.03)
Third level degree	100,762 (69.73)	16,054 (11.11)	19,544 (13.53)	7,551 (5.23)	585 (0.99)
Masters/PhD	31,386 (66.58)	6,184 (13.12)	6,469 (13.72)	2,726 (5.78)	377 (1.46)
Unknown	31,966 (77.36)	3,030 (7.33)	4,518 (10.93)	1,571 (3.80)	235 (1.52)
**Mother's gross income**					
Highest quartile	101,764 (68.19)	16,644 (11.15)	21,382 (14.33)	8,669 (5.81)	781 (1.46)
Second highest quartile	266,390 (72.75)	34,514 (9.43)	46,978 (12.83)	17,275 (4.72)	1,000 (0.99)
Second lowest quartile	121,491 (74.01)	15,421 (9.39)	19,409 (11.82)	7,243 (4.41)	583 (1.12)
Lowest quartile	108,911 (76.36)	12,497 (8.76)	15,344 (19.76)	5,408 (3.79)	475 (1.00)
Unknown	8,696 (80.39)	751 (6.94)	978 (9.04)	355 (3.28)	37 (0.89)
**Father's gross income**					
Highest quartile	271,052 (71.51)	37,426 (9.87)	49,679 (13.11)	19,451 (5.13)	1,454 (1.28)
Second highest quartile	184,028 (73.31)	23,509 (9.36)	31,398 (12.51)	11,398 (4.54)	702 (0.93)
Second lowest quartile	77,164 (74.53)	9,983 (9.64)	11,838 (11.43)	4,235 (4.09)	308 (0.89)
Lowest quartile	59,512 (76.01)	7,227 (9.23)	8,367 (10.69)	2,926 (3.74)	263 (1.01)
Unknown	15,496 (73.52)	1,682 (7.98)	2,809 (13.33)	940 (4.46)	149 (1.84)
**Mother's marital status**					
Married	201,354 (71.91)	26,444 (9.44)	36,330 (12.97)	14,759 (5.27)	1,137 (1.40)
Divorced	13,196 (67.40)	1,804 (9.21)	3,168 (16.18)	1,262 (6.45)	148 (2.55)
Co-habiting	380,426 (73.47)	50,469 (9.75)	63,006 (12.17)	22,364 (4.32)	1,522 (0.92)
Widow	489 (69.46)	44 (6.25)	121 (17.19)	46 (6.53)	4 (2.29)
Unknown	11,787 (79.09)	1,066 (7.15)	1,466 (9.84)	519 (3.48)	65 (1.09)
**Co-morbidities**					
Diabetes	1,882 (42.53)	418 (9.45)	1,219 (27.55)	864 (19.53)	42 (2.45)
Gestational diabetes	90 (27.69)	41 (12.62)	118 (36.31)	74 (22.77)	2 (1.05)
Placenta praevia	638 (26.04)	126 (5.14)	874 (35.67)	803 (32.78)	9 (0.73)
Placental abruption	1,854 (35.20)	257 (4.88)	2,523 (47.90)	624 (11.85)	9 (0.62)
Hypertensive disorders	21,980 (50.75)	4,951 (11.43)	11,833 (27.44)	4,361 (10.07)	135 (0.88)
**Infant birth weight (median [IQR])**	3,400 (3,100, 3,725)	3,546 (3,214, 3,864)	3,450 (2,910, 3,900)	3,200 (2,760, 3,570)	3,370 (3,050, 3,694)
**Infant length, in centimetres (median [IQR])**	52 (50, 53)	52 (51, 54)	52 (50, 54)	50 (49, 52)	51 (50, 52)
**Infant sex**					
Female	303,120 (74.74)	34,514 (8.51)	46,505 (11.47)	20,021 (4.94)	1,379 (1.10)
Male	304,132 (71.15)	45,313 (10.60)	57,586 (13.47)	18,929 (4.43)	1,497 (1.13)
**Gestation, in weeks (mean [SD])**	39.97 (1.85)	40.36 (1.58)	39.44 (2.95)	38.42 (2.48)	38.84 (1.54)
**Gestation**					
Very preterm (<34 wk)	4,702 (41.15)	155 (1.36)	4,847 (42.42)	1,711 (14.97)	12 (0.29)
Preterm (<37 wk)	29,678 (55.75)	2,324 (4.37)	14,853 (27.90)	6,219 (11.68)	157 (0.83)
Term (37–41 wk)	408,026 (75.28)	48,147 (8.88)	53,451 (9.86)	29,852 (5.51)	2,515 (0.46)
Post-term (41+ wk)	159,881 (71.05)	28,482 (12.66)	33,962 (15.09)	2,510 (1.12)	198 (0.29)

Data are *n* (percent) unless otherwise specified. All pregnancy- and infant-related characteristics are for the first (index) live birth.

*Maternally requested cesarean data available from 2002–2010 only (cohort *n* = 258,445).

a Smoking data available 1997–2010 only (*n* = 405,498).

b BMI data available 2003–2010 only (*n* = 230,066).

c History of pregnancy loss before the first live birth.

IQR, interquartile range; SD, standard deviation.

### Missing Data

Variables with missing data included birth weight (*n* = 6,486), maternal BMI (*n* = 41,663), maternal educational attainment (*n* = 41,320), gestational age (*n* = 12,741), father's income (*n* = 21,076), mother's income (*n* = 10,817), mother's marital status (*n* = 14,903), and maternal smoking (*n* = 26,900) (Figure 1). Missing values in the cohort affected potential key covariates. Where a variable had missing data, the variable was re-coded to include missing data as a separate category (for example, for maternal smoking, 1 = smoker, 2 = non-smoker, 3 = missing) and included in the various analyses. As outlined by Vach and Blettner, adding missing data as a separate category where the proportion of missing data is small should not impact greatly on the effect estimates [39].

### Statistical Analysis

The rate of subsequent stillbirth, miscarriage, or ectopic pregnancy was compared by mode of delivery using time-to-event analyses including Cox proportional hazards modelling to estimate the HR and corresponding 95% CIs. In this study, women were followed up from the date of birth of the first child until the subsequent reproductive event of interest (stillbirth, miscarriage, or ectopic pregnancy) or until censoring due to live birth, death, emigration, or study end (December 31, 2010). Separate models were generated for each outcome. For example, when looking at the rate of stillbirth, censoring occurred for live birth, death, emigration, or study end. This was similarly the case for miscarriage and ectopic pregnancy.

### Primary Analyses

Crude and adjusted analyses for all cesarean sections combined compared to operative vaginal delivery and SVD were performed first, followed by analyses including the indication for cesarean section. For maternally requested cesarean section, the cohort was restricted to 2002–2010 for the Cox regression (as maternally requested cesarean was recorded only from 2002 onwards). A priori adjusted analyses comprised three models. Model 1 adjusted for key potential confounders: maternal age, country of origin, obstetric history, maternal educational attainment, mother's marital status, mother's and father's gross income, and birth year. Model 2 adjusted for the covariates in Model 1 plus medical complications of the first live birth including delivery type, diabetes, gestational diabetes, placenta praevia, placental abruption, and hypertensive disorders. Model 3 adjusted for the covariates in Model 2 plus gestational age and birth weight in the first delivery.

### Additional Adjusted and Exploratory Analyses

For each outcome, further adjusted analyses were conducted adding variables to each model for which data were available only for specific time periods: maternal smoking (data from 1997–2010), maternal BMI (data from 2003–2010), and previous fertility treatment (data from 1994–2005). Known or suggested risk factors specific to each outcome were examined in exploratory analyses. For stillbirth, these analyses included a cause-specific competing risks analysis for explained and unexplained stillbirth (data from 1982–1996 only); analyses with data restricted to include mothers who smoked only, term deliveries only, preterm deliveries only, or post-term deliveries only; and analyses to test for the effect over time, in which the cohort was split into three time periods (1982–1991, 1992–2001, and 2002–2010) to assess temporal changes. Finally, as the definition of stillbirth changed from deaths at 28 wk gestation or later to deaths after 22 wk from 2004 onwards, we restricted the data to the time period 2004–2010 to assess whether this had any impact on the findings.

Exploratory analyses for miscarriage included restriction to mothers who smoked only, women of advanced maternal age only (>35 y), fathers of very advanced paternal age only (>45 y), or underweight women only (BMI<18.5 kg/m^2^), and the cohort being split into three time periods as described for stillbirths. Lastly, as the definition of miscarriage also changed from deaths before 28 wk gestation to deaths before 22 wk from 2004 onwards, we restricted the data to the time period 2004–2010 to assess whether this had any impact on the findings.

Exploratory analyses specific to ectopic pregnancy included restriction to women aged 35 y and older only and mothers who smoked only, and testing for a cohort effect. A sensitivity analysis excluding women with a history of pregnancy loss (prior stillbirth, miscarriage, or ectopic pregnancy) was also performed.

Two final exploratory analyses were conducted. First, to check for informative censoring due to live birth, follow-up was limited to 3 y following the primary live birth, and the overall models were re-run for all three outcomes. Second, using multinomial logistic regression, we examined the effect of mode of delivery on subsequent pregnancy outcome in women with a recorded second pregnancy event (i.e., only women with a further stillbirth, miscarriage, ectopic pregnancy, or live birth following the index live birth were included in the analysis). Live birth was the reference group in this composite outcome comparing the odds of pregnancy failure (stillbirth, miscarriage, and ectopic pregnancy) simultaneously.

### Absolute Risk Increase and the Number Needed to Harm

The absolute risk increase (ARI) and the number needed to harm (NNH) were calculated for each outcome according to Barratt et al. [40], where the ARI is equal to the control event rate (CER) minus the experimental event rate (EER):
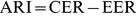
(1)The NNH was calculated as follows by converting the HR:
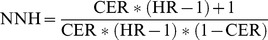
(2)


### Population Attributable Risk

The population attributable risk (PAR) was calculated for each outcome according to a formula outlined by Bruzzi et al. [41]:
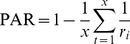
(3)All analyses were performed using SAS version 9.3 software (SAS Institute).

## Results

A summary of demographic, maternal, and infant characteristics according to mode of delivery are presented in Table 1. There were 832,996 first live births, of which 607,252 (72.9%) were SVDs, 79,827 (9.6%) were operative vaginal deliveries, 104,091 (12.5%) were emergency cesareans, 38,950 (4.7%) were elective cesareans, and 2,876 (1.1%) were maternally requested cesareans. The cesarean section rate among first births in 1982 was 12.8%, increasing to 22.8% of first births in 2010. The cesarean section rate for the entire study population over the 28-y follow-up was 17.5%. During the study period, there were 1,996 stillbirths, 73,406 spontaneous miscarriages, and 11,877 ectopic pregnancies (Figure 1).

### Stillbirth

Of the 832,996 women in the cohort, 1,996 had a subsequent stillbirth, a rate of 2.4 per 1,000. The causes of stillbirth were categorized into explained and unexplained (Table 2). An increased rate of stillbirth was found in women with a prior cesarean section (all cesareans combined) in the crude as well as the adjusted analyses (Model 3, HR 1.14, 95% CI 1.01, 1.28) compared to women with a prior SVD (Table 3), giving a theoretical ARI of 0.03% for stillbirth, and a NNH of 3,333 women. Sub-group analyses by indication for cesarean section were similar (emergency cesarean, HR 1.15, 95% CI 1.01, 1.31, and elective cesarean, HR 1.11, 95% CI 0.91, 1.35). There was no increased rate of stillbirth in women with a prior operative vaginal delivery (HR 1.05, 95% CI 0.89, 1.23) compared to women with a prior SVD (Table 3).

**Table 2:** Causes of stillbirth by mode of delivery (1982–1996).

Type of Stillbirth	Previous Delivery	
	Vaginal	Cesarean
**Explained stillbirth (** ***n*** ** = 675)**		
Antenatal complications	79 (18.8)	342 (81.2)
Complications of delivery	35 (24.5)	108 (75.5)
Congenital malformations	10 (11.2)	79 (88.8)
Maternal illness	5 (26.3)	14 (73.7)
Injury to mother	0 (0.00)	3 (100)
**Unexplained stillbirth (** ***n*** ** = 186)**	31 (16.7)	155 (83.3)
**Total**	160 (18.6)	701 (81.4)

Data are *n* (percent). Type of stillbirth was classified as outlined by King-Hele et al. [38].

**Table 3:** Cesarean section and rate of subsequent stillbirth, miscarriage, or ectopic pregnancy.

Outcome by Mode of Delivery^a^	Crude Model HR (95% CI)	Adjusted HR (95% CI)		
		Model 1^b^	Model 2^c^	Model 3^d^
**Stillbirth (** ***n*** ** = 1,996 events)**				
Spontaneous vaginal (*n* = 1,399)	Ref	Ref	Ref	Ref
Operative vaginal (*n* = 172)	1.03 (0.88, 1.20)	1.06 (0.90, 1.25)	1.03 (0.88, 1.22)	1.05 (0.89, 1.23)
All cesarean sections^e^ (*n* = 425)	1.17 (1.05, 1.30)	1.25 (1.12, 1.39)	1.20 (1.07, 1.34)	1.14 (1.01, 1.28)
Emergency cesarean (*n* = 305)	1.19 (1.05, 1.34)	1.25 (1.11, 1.42)	1.21 (1.06, 1.37)	1.15 (1.01, 1.31)
Elective cesarean* (*n* = 120)	1.12 (0.93, 1.35)	1.23 (1.02, 1.49)	1.18 (0.97, 1.43)	1.11 (0.91, 1.35)
**Miscarriage (** ***n*** ** = 73,406 events)**				
Spontaneous vaginal (*n* = 53,540)	Ref	Ref	Ref	Ref
Operative vaginal (*n* = 7,105)	1.09 (1.07, 1.12)	1.03 (1.01, 1.06)	1.02 (1.00, 1.05)	1.02 (1.00, 1.05)
All cesarean sections^e^ (*n* = 12,761)	0.94 (0.92, 0.95)	0.94 (0.92, 0.96)	0.98 (0.96, 1.00)	0.98 (0.96, 1.00)
Emergency cesarean (*n* = 9,178)	0.95 (0.93, 0.98)	0.96 (0.94, 0.98)	0.99 (0.97, 1.01)	0.99 (0.96, 1.01)
Elective cesarean (*n* = 3,453)	0.89 (0.86, 0.92)	0.90 (0.87, 0.93)	0.96 (0.93, 0.99)	0.95 (0.92, 0.98)
Maternally requested cesarean (*n* = 130)	0.68 (0.58, 0.81)	0.69 (0.58, 0.82)	0.72 (0.60, 0.85)	0.72 (0.60, 0.85)
**Ectopic pregnancy (** ***n*** ** = 11,877 events)**				
Spontaneous vaginal (*n* = 8,599)	Ref	Ref	Ref	Ref
Operative vaginal (*n* = 996)	0.96 (0.90, 1.03)	1.02 (0.95, 1.09)	1.02 (0.95, 1.09)	1.03 (0.96, 1.10)
All cesarean sections^e^ (*n* = 2,282)	1.03 (0.98, 1.08)	1.07 (1.02, 1.12)	1.09 (1.03, 1.14)	1.09 (1.04, 1.15)
Emergency cesarean (*n* = 1,605)	1.03 (0.97, 1.08)	1.06 (1.00, 1.12)	1.07 (1.02, 1.13)	1.09 (1.03, 1.15)
Elective cesarean (*n* = 653)	1.03 (0.95, 1.11)	1.09 (1.00, 1.18)	1.12 (1.03, 1.21)	1.12 (1.03, 1.21)
Maternally requested cesarean (*n* = 24)	0.89 (0.60, 1.33)	0.97 (0.65, 1.46)	1.02 (0.68, 1.53)	1.02 (0.68, 1.53)

Data available from 1982–2010 (cohort size = 832,996 women).

a Number of events of the outcome of interest for each mode of delivery in parentheses.

b Model 1: adjusted for maternal age; maternal origin; previous stillbirth, miscarriage, or ectopic pregnancy; mother's marital status; birth year; and measures of socio-economic status including mother's educational attainment and mother's and father's gross income.

c Model 2: adjusted for Model 1 plus medical complications in the first live birth including delivery type (singleton versus twins or more), diabetes, gestational diabetes, placental abruption, placenta praevia, and hypertensive disorders (including eclampsia and preeclampsia).

d Model 3: adjusted for Model 2 plus gestational age and birth weight.

e All emergency, elective, and maternally requested cesarean sections (where applicable, i.e., from 2002–2010) combined.

*Where the number of events was less than ten for maternally requested cesarean, these were combined with the elective cesarean group for analyses.

### Additional Analyses—Explained and Unexplained Stillbirth

A competing risks analysis by cause of stillbirth (Table 4) found an increased rate of explained stillbirth in crude and adjusted models for all cesarean sections combined (HR 1.10, 95% CI 0.89, 1.35) and for prior operative vaginal delivery (HR 1.31, 95% CI 0.84, 2.03); however, neither reached statistical significance. Further analyses by type of cesarean section found an increased rate of explained stillbirth with prior emergency cesarean (HR 1.10, 95% CI 0.87, 1.39) and elective cesarean (HR 1.09, 95% CI 0.76, 1.57); however, both were statistically nonsignificant.

**Table 4:** Cesarean section and rate of subsequent explained or unexplained stillbirth.

Outcome by Mode of Delivery^a^	Crude Model HR (95% CI)	Adjusted HR (95% CI)		
		Model 1^b^	Model 2^c^	Model 3^d^
**Explained stillbirths** e **(** ***n*** ** = 675 events)**				
Spontaneous vaginal (*n* = 520)	Ref	Ref	Ref	Ref
Operative vaginal (*n* = 26)	1.15 (0.77, 1.72)	1.33 (0.86, 2.06)	1.28 (0.83, 1.98)	1.31 (0.84, 2.03)
All cesarean sections (*n* = 129)	1.21 (1.00, 1.47)	1.32 (1.09, 1.61)	1.20 (0.98, 1.47)	1.10 (0.89, 1.35)
Emergency cesarean (*n* = 93)	1.23 (0.98, 1.53)	1.31 (1.05, 1.63)	1.20 (0.96, 1.51)	1.10 (0.87, 1.39)
Elective cesarean* (*n* = 36)	1.17 (0.83, 1.67)	1.37 (0.97, 1.96)	1.21 (0.84, 1.73)	1.09 (0.76, 1.57)
**Unexplained stillbirths** e **(** ***n*** ** = 186 events)**				
Spontaneous vaginal (*n* = 146)	Ref	Ref	Ref	Ref
Operative vaginal (*n* = 9)	1.35 (0.68, 2.65)	0.98 (0.48, 2.00)	0.97 (0.48, 1.98)	0.96 (0.47, 1.96)
All cesarean sections (*n* = 31)	1.08 (0.73, 1.59)	1.17 (0.79, 1.72)	1.13 (0.75, 1.68)	1.14 (0.76, 1.71)
Emergency cesarean (*n* = 26)	1.24 (0.82, 1.89)	1.33 (0.88, 2.03)	1.30 (0.85, 2.00)	1.30 (0.84, 2.01)
Elective cesarean* (*n* = 5)	0.64 (0.26, 1.55)	0.70 (0.29, 1.71)	0.64 (0.26, 1.60)	0.65 (0.27, 1.65)
**All stillbirths 1982–1996 (** ***n*** ** = 861 events)**				
Spontaneous vaginal (*n* = 666)	Ref	Ref	Ref	Ref
Operative vaginal (*n* = 35)	0.81 (0.65, 1.02)	1.03 (0.81, 1.30)	1.00 (0.79, 1.27)	1,01 (0.89, 1.28)
All cesarean sections (*n* = 160)	1.09 (0.96, 1.24)	1.22 (1.07, 1.39)	1.15 (1.01, 1.32)	1.08 (0.94, 1.25)
Emergency cesarean (*n* = 119)	1.14 (0.99, 1.32)	1.25 (1.08, 1.45)	1.20 (1.03, 1.39)	1.13 (0.97, 1.32)
Elective cesarean* (*n* = 41)	0.95 (0.75, 1.20)	1.13 (0.90, 1.43)	1.04 (0.82, 1.32)	0.96 (0.75, 1.23)
**All stillbirths 1997–2010 (** ***n*** ** = 777 events)**				
Spontaneous vaginal (*n* = 496)	Ref	Ref	Ref	Ref
Operative vaginal (*n* = 97)	1.04 (0.84, 1.29)	1.07 (0.86, 1.34)	1.05 (0.84, 1.30)	1.07 (0.86, 1.33)
All cesarean sections (*n* = 184)	1.10 (0.93, 1.30)	1.15 (0.97, 1.36)	1.14 (0.95, 1.36)	1.08 (0.90, 1.30)
Emergency cesarean (*n* = 133)	1.11 (0.92, 1.35)	1.15 (0.95, 1.40)	1.13 (0.93, 1.38)	1.07 (0.87, 1.31)
Elective cesarean* (*n* = 51)	1.05 (0.79, 1.40)	1.13 (0.85, 1.52)	1.15 (0.86, 1.55)	1.11 (0.83, 1.50)

Cohort 1982–1996 (*n* = 427,498 women); cohort 1997–2010 (*n* = 405,498 women).

a Number of events of the outcome of interest for each mode of delivery in parentheses.

b Model 1: adjusted for maternal age; maternal origin; previous stillbirth, miscarriage, or ectopic pregnancy; mother's marital status; birth year; and measures of socio-economic status including mother's educational attainment and mother's and father's gross income.

c Model 2: adjusted for Model 1 plus medical complications in the first live birth including delivery type (singleton versus twins or more), diabetes, gestational diabetes, placental abruption, placenta praevia, and hypertensive disorders (including eclampsia and preeclampsia).

d Model 3: adjusted for Model 2 plus gestational age and birth weight.

e Explained and unexplained stillbirth refer to a cause-specific competing risks for each type of stillbirth. Data for the causes of stillbirth were available from 1982–1996 only (cohort size = 427,498).

*Where the number of events was less than ten for maternally requested cesarean, these were combined with the elective cesarean group for analyses.

An increased rate of unexplained stillbirth was found among all cesarean sections combined (HR 1.14, 95% CI 0.76, 1.71); however, this was not statistically significant (Table 4). No increased rate of unexplained stillbirth was found among prior operative vaginal delivery (HR 0.96, 95% CI 0.47, 1.96). When the indication for cesarean section was analysed, an increased rate of unexplained stillbirth was found for prior emergency cesarean only (HR 1.30, 95% CI 0.84, 2.01), although this did not reach significance. Prior elective cesarean (HR 0.65, 95% CI 0.27, 1.65) was not associated with any increased rate of unexplained stillbirth.

Further comparative analyses for all stillbirths during the time period when data on the causes of stillbirth existed (1982–1996) found a similar rate among all cesarean sections (HR 1.08, 95% CI 0.94, 1.25) and by type of cesarean section, with prior emergency cesarean section again showing an increased rate (HR 1.13, 95% CI 0.97, 1.32). Looking at the later time period (1997–2010), all cesarean sections were associated with an increased rate of stillbirth (HR 1.08, 95% CI 0.90, 1.30), as were emergency cesarean section (HR 1.07, 95% CI 0.87, 1.31) and elective cesarean section (HR 1.11, 95% CI 0.83, 1.50), although none reached conventional statistical significance (Table 4).

Further adjusted analyses (by maternal smoking, maternal BMI, and previous fertility treatment) and sub-group analyses (restricted to mothers who smoked, term deliveries, preterm deliveries, and post-term deliveries) are described in Text S1, and the results are presented in Table S1.

### Miscarriage

Of the 832,996 women in the cohort, 73,406 had a subsequent miscarriage, a rate of 8.8 per 100. When all cesarean sections were combined, no significantly increased rate of miscarriage was found (HR 0.98, 95% CI 0.96, 1.00). When the data were analysed separately by indication for cesarean section, no increased rate of miscarriage was found for previous emergency cesarean (HR 0.99, 95% CI 0.96, 1.01) or elective cesarean (HR 0.95, 95% CI 0.92, 0.98). Maternally requested cesarean section (HR 0.72, 95% CI 0.60, 0.85) was associated with a decreased rate of subsequent miscarriage (Table 3).

### Additional Analyses—Miscarriage

Further adjusted analyses (by maternal smoking, maternal BMI, and previous fertility treatment) and sub-group analyses (restricted to mothers who smoked, advanced paternal age, and underweight maternal BMI) are detailed in Text S1, and the results are presented in Table S2.

### Ectopic Pregnancy

Of the 832,996 women in the cohort, 11,877 had a subsequent ectopic pregnancy, a rate of 1.4 per 100 women. When all cesareans were combined, a 9% increased rate of ectopic pregnancy was found (HR 1.09, 95% CI 1.04, 1.15), yielding an ARI of 0.1% and a NNH of 1,000 women. When cesareans were analysed according to indication (Table 3), an increased rate of subsequent ectopic pregnancy was found in women with a prior emergency cesarean (HR 1.09, 95% CI 1.03, 1.15) and elective cesarean (HR 1.12, 95% CI 1.03, 1.21).

### Additional Analyses—Ectopic Pregnancy

Further adjusted analyses (by previous fertility treatment) and sub-group analyses (restricted to women who smoked and advanced maternal age) are described in Text S1. The results are presented in Table S3.

### Additional Analyses—All Outcomes

A sensitivity analysis excluding women with a history of pregnancy loss as described in Text S1 and Table S4 did not change the overall conclusions. When the cohort was restricted to a maximum of 3 y of follow-up, the results overall did not change significantly (Table 5). When the odds of pregnancy failure were examined simultaneously in a multinomial logistic regression (Table 6), overall there was a significantly increased odds of subsequent stillbirth, miscarriage, and ectopic pregnancy among women with a prior cesarean section.

**Table 5:** Cesarean section and rate of subsequent stillbirth, miscarriage, or ectopic pregnancy—3-y follow-up.

Outcome by Mode of Delivery^a^	Crude Model HR (95% CI)	Adjusted HR (95% CI)		
		Model 1^b^	Model 2^c^	Model 3^d^
**Stillbirth (** ***n*** ** = 916 events)**				
Spontaneous vaginal (*n* = 632)	Ref	Ref	Ref	Ref
Operative vaginal (*n* = 83)	0.97 (0.77, 1.22)	0.96 (0.76, 1.21)	0.94 (0.74, 1.19)	0.96 (0.76, 1.22)
All cesarean sections**^e^** (*n* = 201)	1.26 (1.08, 1.48)	1.30 (1.11, 1.53)	1.25 (1.06, 1.48)	1.18 (1.00, 1.40)
Emergency cesarean (*n* = 148)	1.30 (1.09, 1.56)	1.34 (1.12, 1.61)	1.29 (1.07, 1.56)	1.22 (1.01, 1.47)
Elective cesarean* (*n* = 53)	1.17 (0.88, 1.55)	1.20 (0.91, 1.60)	1.15 (0.86, 1.54)	1.08 (0.81, 1.45)
**Miscarriage (** ***n*** ** = 45,321 events)**				
Spontaneous vaginal (*n* = 32,883)	Ref	Ref	Ref	Ref
Operative vaginal (*n* = 4,780)	1.11 (1.07, 1.14)	1.02 (0.99, 1.05)	1.01 (0.98, 1.04)	1.09 (1.06, 1.13)
All cesarean sections^e^ (*n* = 7,554)	0.94 (0.91, 0.96)	0.92 (0.90, 0.94)	0.95 (0.93, 0.98)	0.95 (0.93, 0.98)
Emergency cesarean (*n* = 5,519)	0.95 (0.92, 0.98)	0.94 (0.91, 0.97)	0.96 (0.93, 0.99)	0.96 (0.93, 0.99)
Elective cesarean (*n* = 2,035)	0.91 (0.87, 0.95)	0.87 (0.84, 0.91)	0.93 (0.89, 0.97)	0.93 (0.89, 0.97)
Maternally requested cesarean (*n* = 104)	0.68 (0.56, 0.82)	0.70 (0.58, 0.85)	0.73 (0.60, 0.88)	0.78 (0.64, 0.95)
**Ectopic pregnancy (** ***n*** ** = 6,278 events)**				
Spontaneous vaginal (*n* = 4,467)	Ref	Ref	Ref	Ref
Operative vaginal (*n* = 596)	1.01 (0.93, 1.10)	1.02 (0.93, 1.11)	1.01 (0.93, 1.11)	1.06 (0.97, 1.15)
All cesarean sections^e^ (*n* = 1,215)	1.10 (1.03, 1.17)	1.08 (1.01, 1.15)	1.08 (1.02, 1.16)	1.08 (1.01, 1.16)
Emergency cesarean (*n* = 893)	1.13 (1.05, 1.21)	1.11 (1.03, 1.20)	1.12 (1.04, 1.20)	1.12 (1.04, 1.21)
Elective cesarean* (*n* = 322)	1.02 (0.91, 1.14)	0.99 (0.88, 1.11)	1.00 (0.89, 1.12)	0.99 (0.88, 1.11)

Women were followed up for a maximum of 3 y from the date of the first live birth until the subsequent event to test for informative censoring due to live birth. Cohort 1982–2010 (*n* = 832,996 women).

a Number of events of the outcome of interest for each mode of delivery in parentheses.

b Model 1: adjusted for maternal age; maternal origin; previous stillbirth, miscarriage, or ectopic pregnancy; mother's marital status; birth year; and measures of socio-economic status including mother's educational attainment and mother's and father's gross income.

c Model 2: adjusted for Model 1 plus medical complications in the first live birth including delivery type (singleton versus twins or more), diabetes, gestational diabetes, placental abruption, placenta praevia, and hypertensive disorders (including eclampsia and preeclampsia).

d Model 3: adjusted for Model 2 plus gestational age and birth weight.

*Where the number of events was less than ten for maternally requested cesarean, these were combined with the elective cesarean group for analyses.

**Table 6:** Cesarean section and the odds of stillbirth, miscarriage, or ectopic pregnancy—a multinomial logistic regression analysis.

Outcome by Mode of Delivery^a^	Crude Model OR (95% CI)	Adjusted OR (95% CI)		
		Model 1^b^	Model 2^c^	Model 3^d^
**Stillbirth (** ***n*** ** = 1,518 events)**				
Spontaneous vaginal (*n* = 1,074)	Ref	Ref	Ref	Ref
Operative vaginal (*n* = 123)	0.93 (0.77, 1.12)	0.98 (0.81, 1.18)	0.96 (0.79, 1.17)	0.99 (0.81, 1.20)
All cesarean sections (*n* = 321)	1.54 (1.36, 1.74)	1.50 (1.32, 1.71)	1.40 (1.23, 1.60)	1.32 (1.16, 1.51)
Emergency cesarean (*n* = 231)	1.50 (1.30, 1.73)	1.48 (1.28, 1.71)	1.39 (1.20, 1.61)	1.32 (1.13, 1.53)
Elective cesarean* (*n* = 90)	1.64 (1.32, 2.04)	1.57 (1.26, 1.96)	1.43 (1.15, 1.79)	1.34 (1.07, 1.68)
**Miscarriage (** ***n*** ** = 68,985 events)**				
Spontaneous vaginal (*n* = 50,363)	Ref	Ref	Ref	Ref
Operative vaginal (*n* = 6,728)	1.09 (1.06, 1.12)	1.03 (1.00, 1.06)	1.03 (1.00, 1.06)	1.04 (1.01, 1.07)
All cesarean sections (*n* = 11,894)	1.21 (1.19, 1.24)	1.15 (1.12, 1.17)	1.13 (1.11, 1.16)	1.12 (1.09, 1.14)
Emergency cesarean (*n* = 8,565)	1.19 (1.16, 1.22)	1.13 (1.11, 1.16)	1.12 (1.09, 1.15)	1.11 (1.08, 1.14)
Elective cesarean (*n* = 3,206)	1.28 (1.24, 1.33)	1.19 (1.15, 1.24)	1.17 (1.12, 1.22)	1.14 (1.10, 1.19)
Maternally requested cesarean (*n* = 123)	1.37 (1.13, 1.66)	1.12 (0.92, 1.37)	1.12 (0.92, 1.36)	1.11 (0.91, 1.36)
**Ectopic pregnancy (** ***n*** ** = 11,877 events)**				
Spontaneous vaginal (*n* = 8,599)	Ref	Ref	Ref	Ref
Operative vaginal (*n* = 996)	0.94 (0.88, 1.01)	1.02 (0.95, 1.09)	1.02 (0.96, 1.10)	1.04 (0.97, 1.12)
All cesarean sections (*n* = 2,282)	1.37 (1.30, 1.43)	1.34 (1.28, 1.41)	1.29 (1.23, 1.36)	1.30 (1.23, 1.36)
Emergency cesarean (*n* = 1,605)	1.31 (1.24, 1.38)	1.29 (1.22, 1.36)	1.25 (1.18, 1.32)	1.26 (1.19, 1.33)
Elective cesarean (*n* = 653)	1.53 (1.41, 1.66)	1.50 (1.38, 1.63)	1.42 (1.31, 1.55)	1.39 (1.28, 1.52)
Maternally requested cesarean (*n* = 24)	1.79 (1.19, 2.70)	1.62 (1.05, 2.50)	1.60 (1.04, 2.47)	1.59 (1.03, 2.46)

Cohort 1982–2010 (*n* = 832,996 women).

a Number of events of the outcome of interest for each mode of delivery in parentheses. Outcome refers to a composite outcome whereby the odds of pregnancy failure (stillbirth, miscarriage, or ectopic pregnancy) were compared simultaneously using multinomial logistic regression models. The reference group was live birth (*n* = 514,191). Women with no recorded second pregnancy event (*n* = 236,425) were not included in this analysis.

b Model 1: adjusted for maternal age; maternal origin; previous stillbirth, miscarriage, or ectopic pregnancy; mother's marital status; birth year; and measures of socio-economic status including mother's educational attainment and mother's and father's gross income.

c Model 2: adjusted for Model 1 plus medical complications in the first live birth including delivery type (singleton versus twins or more), diabetes, gestational diabetes, placental abruption, placenta praevia, and hypertensive disorders (including eclampsia and preeclampsia).

d Model 3: adjusted for Model 2 plus gestational age and birth weight.

*Where the number of events was less than ten for maternally requested cesarean, these were combined with the elective cesarean group for analyses.

### PAR and PAR Percent

#### Stillbirth PAR calculations

Attributable risk (AR) numbers are taken from Table 3 (estimates for adjusted Model 3 by indication for mode of delivery):
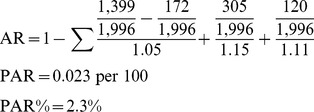
(4)


#### Ectopic pregnancy PAR calculations

AR numbers are taken from Table 3. Note that for group 5 (maternally requested cesarean) (asterisk), data are available only from 2002, and the total population number of events differs as a result.
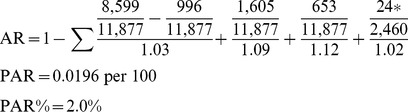
(5)


## Discussion

The results show that women with a prior cesarean section had an increased hazard of subsequent stillbirth by 14%, and the hazard increase was similar for both elective (11%) and emergency (15%) cesarean sections, although this increase did not reach statistical significance among elective cesarean sections. No significantly increased hazard of stillbirth was found in women with a prior operative vaginal delivery. A competing risks analysis for the causes of stillbirth showed a 10% increased rate of explained stillbirth among all women with a prior cesarean section. Analyses by indication for cesarean produced an equally increased hazard among women with a prior emergency cesarean (10%) and a prior elective cesarean delivery (9%); however, neither reached statistical significance. A 14% increased hazard of unexplained stillbirth for all women with a prior cesarean section was found, which increased to 30% for women with a prior emergency cesarean section in the analysis by indication, although neither result reached significance. Elective cesarean and operative vaginal delivery appeared to have a decreased rate of subsequent unexplained stillbirth; however, there were only nine unexplained stillbirths in the operative vaginal group and five unexplained stillbirths in the elective cesarean group between 1982 and 1996.

Almost 50% of the explained stillbirths in this cohort were due to antenatal complications (including placental abruption/infarction, intrauterine growth restriction, preeclampsia, prematurity, and poor placental growth). The clinical importance is that although many of these complications are largely not preventable, an increased awareness that the fetus is at risk may facilitate increased surveillance and optimally timed delivery and may lead to improved perinatal outcomes [42]. These results show that there were changes in the hazard rate of stillbirth over time. An increased rate of stillbirth was apparent for prior emergency cesarean section in the earlier time periods (1982–1991 and 1992–2001), but this rate was driven by undiagnosed medical complications presenting at the time of delivery. This increased rate of stillbirth shifted to elective cesarean section in the more recent years (2002–2010). This increased rate of stillbirth could be due to underlying factors contributing to the need for a cesarean section, and not the cesarean section per se. It is also apparent that the rates of cesarean section among first-time mothers in Denmark increased during the study time period, from 12.8% in 1982 to almost 23% in 2010. This increase, coupled with better surveillance and detection of adverse or underlying complications earlier in pregnancy, could explain the increased hazard of subsequent stillbirth found in this study among women with a prior cesarean section.

There was no increase in the hazard of subsequent miscarriage among all cesarean sections, and maternally requested cesareans appeared to have a decreased rate, by as much as 40%. In the additional multinomial logistic regression analysis, however, there is some evidence of a small increased odds of miscarriage following cesarean section delivery. A small increased hazard rate of ectopic pregnancy was found among women with a prior cesarean section (9%). This hazard remained in the analysis by indication for emergency cesarean (9%) and elective cesarean (12%). This rate increased significantly when maternal BMI, maternal smoking, and previous fertility treatment were added to the models. An increased rate of ectopic pregnancy was found across the three time periods, with the hazard rate peaking in the later years, perhaps in line with the rising cesarean section rate.

The current findings are in agreement with a recent systematic review and meta-analysis exploring the relationship between prior cesarean section delivery and the likelihood of subsequent stillbirth and miscarriage [21], although the review reported a much higher rate of subsequent stillbirth. The meta-analysis found a 23% increased odds of subsequent stillbirth and a 47% increased odds of unexplained stillbirth in women with a prior cesarean section compared to a prior vaginal delivery in ten studies spanning from 1968 to 2009. However, in the systematic review, it was not possible to perform stratified analyses based on the indication for cesarean section. As outlined earlier, it is possible that the increased hazard rate of stillbirth could be due to the underlying reasons for the emergency or elective cesarean sections, and these underlying reasons, rather than the cesarean section itself, may be the driving force behind the increased rates of stillbirth. The findings of an increased rate of stillbirth following cesarean in the current study and in previous studies [20],[43]–[45] are important for expectant mothers, particularly those requesting a cesarean section without any medical indication. On the other hand, it must be acknowledged that a cesarean section can be a vital intervention, and the likelihood of adverse outcome may be decreased, for example, by choosing an elective cesarean section to avoid fetal death due to a failed vaginal birth after cesarean or to prevent sudden stillbirth post-term.

In the recent systematic review [21] it was not possible to include the identified miscarriage studies in a meta-analysis because of poor epidemiological methods, primarily a lack of confounder adjustment. The authors, however, concluded that there was insufficient evidence to determine that cesarean section increased the risk of subsequent miscarriage, in line with the findings of the current study. One of the largest studies to date used retrospective data from the Scottish Morbidity Record [46], and reported that residual confounding could be a plausible reason for any increased hazard of miscarriage reported for women with a prior cesarean in previous studies.

This study found a moderately increased rate of ectopic pregnancy among women with a prior emergency and elective cesarean section, in contrast to the findings of a recent meta-analysis [23], where although a 5% increased odds of ectopic pregnancy was found among the included studies, the estimate did not reach statistical significance. The studies included in the meta-analysis, however, were limited by many factors including small sample size (the total sample included from the five studies was 4,716 women and 490 ectopic pregnancies, compared to over 832,000 women and over 11,800 ectopic pregnancies in the current analysis) and lack of detailed information regarding women's obstetric history and the indication for mode of delivery.

### PAR and PAR Percent

A reduction of 0.023 stillbirths per 100 population is expected if women were not exposed to a cesarean delivery (PAR = 0.023 per 100), representing a 2.3% reduction of the incidence in the population (PAR% = 2.3%). For ectopic pregnancy, a reduction of 0.0196 ectopic pregnancies per 100 population is expected if women were not exposed to a cesarean delivery (PAR = 0.0196 per 100). This represents a 2.0% reduction of the incidence in the population (PAR% = 2.0%).

### Strengths and Limitations

The strengths of the present study include the use of national register-based data and accurate linkage between and within registers with individuals' unique CPR numbers. Such linkage enriches the data available for the present study, including detailed obstetric data on the indication for cesarean section and novel data on maternally requested cesarean section. To our knowledge, this is also the largest study to date investigating the outcomes stillbirth, miscarriage, and ectopic pregnancy with follow-up covering almost three decades and the majority of women's reproductive careers. Extensive analyses adjusting for numerous potential confounders, sub-group analyses, and a competing risks analysis on the causes of stillbirth (categorized into explained and unexplained stillbirths using 15 y of the data) add to the findings. Furthermore, with the large sample size, we were able to perform multiple Cox regression analyses where censoring due to birth was accounted for. This method allowed for the assessment of the rate of a particular outcome taking into consideration the varying duration of pregnancy, as well as the fact that some women will have no further pregnancies.

There are, however, some limitations to the current study. First, we acknowledge the possibility of misclassification or underreporting of the outcomes of interest. We restricted our cohort to include only women who gave birth from 1982 to 2010, however, to ensure that the MBR (established in 1973) and the NHR (established in 1977) would have complete coverage by this date. We also believe that the outcomes of interest, and particularly stillbirth, would not be underreported. Nevertheless, there may be a possibility that some miscarriage cases (particularly very early miscarriages) may have been missed, and this is an acknowledged limitation. Data on miscarriage in the Danish registers have been validated recently and deemed suitable for epidemiological studies. One study found that over 97% of miscarriage diagnoses were correct, although the sample size was small [35]. A second study including over 11,000 women and over 650 spontaneous miscarriages, however, reported that up to 25% of miscarriages may not end up registered (in very early miscarriages where women do not require/seek hospitalisation), so caution is warranted [47]. Second, data on the causes of stillbirth were available only for 15 y of the study, and it was not possible to segregate stillbirths into antepartum and intrapartum stillbirths for the current analysis. Furthermore, the cutoff for defining stillbirth versus miscarriage changed from 28 wk to 22 wk gestation in 2004; however, information on stillbirth and miscarriage gestational age, to confirm correct classification, was not available for the current analyses. For ectopic pregnancy, no data were available on known risk factors, including the number of previous sexual partners, history of pelvic inflammatory disease, and age at first intercourse. Limited availability of data for maternally requested cesarean as well as maternal BMI (2003–2010), maternal smoking (1997–2010), and access to fertility services (1994–2005) and missing data for variables are acknowledged limitations; however, it was possible to test for the potential confounding effects of these variables in sensitivity analyses.

Two statistical methods could have been used to answer the current research question regarding the effect of mode of delivery on the rate of subsequent stillbirth, miscarriage, and ectopic pregnancy. The main analyses in this study included Cox proportional hazards modelling. We also conducted further exploratory analyses, however, where we used multinomial logistic regression modelling to assess the impact of mode of delivery on the odds of subsequent pregnancy failure in women with a recorded second pregnancy event only (i.e., only women with a subsequent live birth, stillbirth, miscarriage, or ectopic pregnancy following the index live birth were included in the analyses). Whilst there is no perfect model to answer this question in the absence of data on time of conception, the Cox model could be considered the less biased option as it takes into account length of follow-up and includes all women, even those who do not go on to have a subsequent pregnancy event. The multinomial logistic regression excludes women who have no further pregnancy event and therefore conditions on the future. Both methods, however, supported similar conclusions, with an increased rate or odds of stillbirth and ectopic pregnancy with prior cesarean section in the models. In the logistic regression, however, a small increased odds of miscarriage was also found, in contrast to the Cox model.

Whilst we were able to assess temporal changes in the current study, with data that spanned almost three decades (from 1982 to 2010), it must be acknowledged that many changes in prenatal and neonatal care, as well as changes in obstetric training and techniques, cesarean section rates, and societal behaviour, may have influenced the results. Residual confounding cannot be ruled out in the present study, as is the case with many epidemiological observational studies. A randomized control trial, however, is unlikely to be ethical or feasible for the current research question in the near future.

### Conclusions

The findings of this study suggest that a prior cesarean section is associated with a small increased rate of subsequent stillbirth and ectopic pregnancy. These are two rare pregnancy outcomes, however, and the increased rate is statistically significant but small in size. No increased rate of miscarriage was found for prior cesarean section, and maternally requested cesarean section appeared to be protective against subsequent miscarriage. The findings of the current study are particularly important for expectant mothers as well as health-care professionals as they highlight that although cesarean section rates are increasing significantly worldwide, there is no dangerously increased rate of subsequent stillbirth, miscarriage, or ectopic pregnancy. Furthermore, the findings will better inform women of the benefits and risks associated with all modes of delivery and help women and their partners make a more informed decision regarding mode of delivery based on their individual pregnancy circumstances. On the other hand, while the hazard rate is small, women “electing” cesarean section without any medical necessity should consider all options thoroughly. Considering the important public health consequences of stillbirth and ectopic pregnancy, further research is warranted using large-scale data as in the current study, and in the absence of clinical trials to confirm the present study findings and add to the current recommendations for the management of pregnancy following cesarean section.

## Supporting Information

Table S1 **Cesarean section and rate of subsequent stillbirth—additional analyses.**
(DOCX)

Table S2 **Cesarean section and rate of subsequent miscarriage—additional analyses.**
(DOCX)

Table S3 **Cesarean section and rate of subsequent ectopic pregnancy—additional analyses.**
(DOCX)

Table S4 **Cesarean section and rate of subsequent stillbirth, miscarriage, or ectopic pregnancy—sensitivity analyses excluding women with a history of pregnancy loss.**
(DOCX)

Text S1 **Cesarean section and rate of subsequent stillbirth, miscarriage, or ectopic pregnancy—additional analyses.**
(DOCX)

## Abbreviations

AR: attributable risk
ARI: absolute risk increase
BMI: body mass index
CPR: civil personal registration
CRS: Danish Civil Registration System
HR: hazard ratio
ICD: International Classification of Diseases
MBR: Danish Medical Birth Registry
NHR: Danish National Hospital Register
NNH: number needed to harm
OR: odds ratio
PAR: population attributable risk
SVD: spontaneous vaginal delivery
