# Correction: Cesarean Section and Rate of Subsequent Stillbirth, Miscarriage, and Ectopic Pregnancy: A Danish Register-Based Cohort Study

**DOI:** 10.1371/journal.pmed.1001707

**Published:** 2014-07-28

**Authors:** The PLOS Medicine Staff

## Abstract

Louise Kenny and colleagues conduct a population-based cohort study in Denmark to assess the likelihood of stillbirth, miscarriage, and ectopic pregnancy following cesarean section compared to women who gave birth by vaginal delivery.

There is an error in Table 2. The headings under ‘Previous Delivery’ are labeled incorrectly; ‘Vaginal’ and ‘Cesarean’ should be in the reverse order, as shown below.

**Table 2:** Causes of stillbirth by mode of delivery (1982–1996).

Type of Stillbirth	Previous Delivery	
	Cesarean	Vaginal
**Explained stillbirth (** ***n = *** ** 675)**		
Antenatal complications	79 (18.8)	342 (81.2)
Complications of delivery	35 (24.5)	108 (75.5)
Congenital malformations	10 (11.2)	79 (88.8)
Maternal illness	5 (26.3)	14 (73.7)
Injury to mother	0 (0.00)	3 (100)
**Unexplained stillbirth (** ***n = *** ** 186)**	31 (16.7)	155 (83.3)
**Total**	160 (18.6)	701 (81.4)

Data are *n* (percent). Type of stillbirth was classified as outlined by King-Hele et al. [38].
