# Supplementary material for: Cesarean Section and Rate of Subsequent Stillbirth, Miscarriage, and Ectopic Pregnancy: A Danish Register-Based Cohort Study
Source: PLoS Med. 2014 Jul 1;11(7):e1001670. doi: 10.1371/journal.pmed.1001670 (PMC4077571; doi:10.1371/journal.pmed.1001670)
Supplement: Table S1 — Cesarean section and rate of subsequent stillbirth—additional analyses. (DOCX) [file pmed.1001670.s001.docx]

**Table S1:** Cesarean section and rate of subsequent stillbirth – additional analyses

| **^a^Mode of delivery** | **Cohort** Varies according to analyses | | | |
| --- | --- | --- | --- | --- |
| **Outcome: Stillbirth** | **Crude Model** | **Adjusted HR (95% CI)** | | |
| Smoking adjustment (data from 1997-2010) (events n=777) | **Cr. HR (95% CI)** | **^b^ Model 1** | **^c^ Model 2** | **^d^ Model 3** |
| Spontaneous vaginal (n=496) | *ref* | *ref* | *ref* | *ref* |
| Operative vaginal (n=97) | 1.04 (0.84, 1.30) | 1.08 (0.86, 1.34) | 1.05 (0.84, 1.31) | 1.07 (0.86, 1.33) |
| Emergency Cesarean (n=133) | 1.11 (0.92, 1.35) | 1.15 (0.95, 1.40) | 1.13 (0.92, 1.37) | 1.07 (0.87, 1.31) |
| *****Elective Cesarean (n=51) | 1.05 (0.79, 1.40) | 1.13 (0.84, 1.51) | 1.15 (0.85, 1.54) | 1.11 (0.82, 1.49) |
| BMI adjustment (data from 2003-2010) (events n=350) | **Cr. HR (95% CI)** | **Model 1** | **Model 2** | **Model 3** |
| Spontaneous vaginal (n=222) | *ref* | *ref* | *ref* | *ref* |
| Operative vaginal (n=42) | 0.93 (0.67, 1.30) | 0.97 (0.70, 1.35) | 0.96 (0.69, 1.34) | 0.98 (0.70, 1.37) |
| Emergency Cesarean (n=60) | 0.97 (0.73, 1.29) | 1.01 (0.76, 1.35) | 1.00 (0.75, 1.34) | 0.96 (0.71, 1.29) |
| *****Elective Cesarean (n=26) | 0.97 (0.73, 1.29) | 1.06 (0.70, 1.60) | 1.09 (0.72, 1.65) | 1.05 (0.69, 1.60) |
| Fertility treatment adjustment (data from 1994-2005) (events n=877) | **Crude Model** | **Model 1** | **Model 2** | **Model 3** |
| Spontaneous vaginal (n=562) | *ref* | *ref* | *ref* | *ref* |
| Operative vaginal (n=115) | 1.04 (0.85, 1.27) | 1.07 (0.88, 1.31) | 1.05 (0.86, 1.29) | 1.07 (0.88, 1.31) |
| Emergency Cesarean (n=141) | 1.18 (0.98, 1.42) | 1.22 (1.01, 1.47) | 1.22 (1.01, 1.47) | 1.16 (0.96, 1.41) |
| *****Elective Cesarean (n=59) | 1.14 (0.87, 1.50) | 1.23 (0.94, 1.61) | 1.23 (0.94, 1.63) | 1.15 (0.87, 1.52) |
| Restricted to smokers only (data from 1997-2010) (events n=150) | **Crude Model** | **Model 1** | **Model 2** | **Model 3** |
| Spontaneous vaginal (n=102) | *ref* | *ref* | *ref* | *ref* |
| Operative vaginal (n=11) | 0.62 (0.33, 1.16) | 0.66 (0.35, 1.23) | 0.64 (0.34, 1.20) | 0.65 (0.35, 1.22) |
| Emergency Cesarean (n=22) | 0.95 (0.60, 1.51) | 1.02 (0.64, 1.63) | 0.94 (0.58, 1.51) | 0.84 (0.51, 1.37) |
| *****Elective Cesarean (n=15) | 1.67 (0.97, 2.87) | 1.82 (1.05, 3.17) | 1.76 (1.00, 3.09) | 1.64 (0.93, 2.90) |
| Restricted to term deliveries (data from 1982-2010) (events n=1,253) | **Crude Model** | **Model 1** | **Model 2** | **Model 3** |
| Spontaneous vaginal (n=918) | *ref* | *ref* | *ref* | *ref* |
| Operative vaginal (n=96) | 0.97 (0.79, 1.20) | 1.01 (0.81, 1.25) | 0.98 (0.78, 1.21) | 0.98 (0.79, 1.22) |
| Emergency Cesarean (n=165) | 1.28 (1.09, 1.51) | 1.34 (1.14, 1.59) | 1.29 (1.09, 1.53) | 1.26 (1.06, 1.50) |
| *****Elective Cesarean (n=74) | 0.94 (0.74, 1.19) | 1.03 (0.81, 1.31) | 1.01 (0.79, 1.29) | 0.99 (0.78, 1.26) |
| Restricted to preterm deliveries (data from 1982-2010) (events n=179) | **Cr. HR (95% CI)** | **Model 1** | **Model 2** | **Model 3** |
| Spontaneous vaginal (n=73) | *ref* | *ref* | *ref* | *ref* |
| Operative vaginal (n=8) | 1.53 (0.74, 3.17) | 1.70 (0.81, 3.58) | 1.55 (0.74, 3.25) | 1.65 (0.78, 3.48) |
| Emergency Cesarean (n=59) | 1.46 (1.04, 2.06) | 1.60 (1.13, 2.26) | 1.55 (1.07, 2.24) | 1.37 (0.94, 1.99) |
| *****Elective Cesarean (n=39) | 2.09 (1.42, 3.09) | 2.40 (1.60, 3.60) | 2.18 (1.40, 3.20) | 1.90 (1.22, 2.98) |

**Table S1:** Cesarean section and rate of subsequent stillbirth – additional analyses (continued)

| **Mode of delivery^a^** | **Cohort** Varies according to analyses | | | |
| --- | --- | --- | --- | --- |
| **Outcome: Stillbirth** | **Crude Model** | **Adj. HR (95% CI)** | | |
| Restricted to post term deliveries (data from 1982-2010) (events n=526) | **Crude Model** | **^b^ Model 1** | **^c^ Model 2** | **^d^ Model 3** |
| Spontaneous vaginal (n=383) | *ref* | *ref* | *ref* | *ref* |
| Operative vaginal (n=58) | 0.93 (0.71, 1.23) | 0.96 (0.72, 1.28) | 0.95 (0.72, 1.27) | 0.96 (0.72, 1.29) |
| Emergency Cesarean (n=78) | 0.94 (0.73, 1.19) | 1.00 (0.78, 1.27) | 0.98 (0.77, 1.26) | 0.99 (0.77, 1.27) |
| *****Elective Cesarean (n=7) | 1.03 (0.49, 2.18) | 1.13 (0.53, 2.39) | 1.11 (0.52, 2.34) | 1.11 (0.53, 2.36) |
| Cohort effect (data restricted to 1982-1991) (events n=776) | **Crude Model** | **Model 1** | **Model 2** | **Model 3** |
| Spontaneous vaginal (n=613) | *ref* | *ref* | *ref* | *ref* |
| Operative vaginal (n=14) | 1.14 (0.67, 1.93) | 1.31 (0.73, 2.34) | 1.26 (0.71, 2.26) | 1.27 (0.71, 2.27) |
| Emergency Cesarean (n=115) | 1.28 (1.05, 1.56) | 1.35 (1.11, 1.65) | 1.25 (1.02, 1.54) | 1.19 (0.97, 1.47) |
| Elective Cesarean (n=34) | 1.05 (0.74, 1.48) | 1.18 (0.83, 1.67) | 1.06 (0.75, 1.51) | 1.04 (0.73, 1.48) |
| Cohort effect (data restricted to 1992-2001) (events n=799) | **Crude Model** | **Model 1** | **Model 2** | **Model 3** |
| Spontaneous vaginal (n=528) | *ref* | *ref* | *ref* | *ref* |
| Operative vaginal (n=108) | 1.01 (0.81, 1.23) | 1.05 (0.85, 1.29) | 1.02 (0.83, 1.25) | 1.04 (0.84, 1.28) |
| Emergency Cesarean (n=113) | 1.12 (0.92, 1.38) | 1.21 (0.99, 1.49) | 1.20 (0.97, 1.47) | 1.13 (0.92, 1.40) |
| Elective Cesarean (n=50) | 1.08 (0.81, 1.44) | 1.22 (0.91, 1.64) | 1.17 (0.86, 1.58) | 1.04 (0.76, 1.41) |
| Cohort effect (data restricted to 2002-2010) ( events n=421) | **Crude Model** | **Model 1** | **Model 2** | **Model 3** |
| Spontaneous vaginal (n=258) | *ref* | *ref* | *ref* | *ref* |
| Operative vaginal (n=50) | 0.96 (0.71, 1.29) | 1.00 (0.74, 1.35) | 0.98 (0.73, 1.33) | 1.00 (0.74, 1.36) |
| Emergency Cesarean (n=77) | 1.10 (0.85, 1.42) | 1.16 (0.90, 1.50) | 1.15 (0.88, 1.49) | 1.09 (0.84, 1.43) |
| *****Elective Cesarean (n=36) | 1.19 (0.84, 1.69) | 1.29 (0.91, 1.84) | 1.32 (0.93, 1.89) | 1.29 (0.90, 1.85) |
| Definition of stillbirth (data restricted to 2004-2010) ( events n=274) | **Crude Model** | **Model 1** | **Model 2** | **Model 3** |
| Spontaneous vaginal (n=176) | *ref* | *ref* | *ref* | *ref* |
| Operative vaginal (n=28) | 0.78 (0.52, 1.16) | 0.81 (0.54, 1.21) | 0.81 (0.54, 1.20) | 0.83 (0.55, 1.24) |
| Emergency Cesarean (n=48) | 0.99 (0.72, 1.36) | 1.04 (0.75, 1.44) | 1.01 (0.73, 1.41) | 0.97 (0.69, 1.36) |
| *****Elective Cesarean (n=22) | 1.06 (0.68, 1.66) | 1.15 (0.74, 1.80) | 1.17 (0.75, 1.85) | 1.13 (0.71, 1.77) |

**Data refer to: Cr. HR:** Crude Hazard Ratio (95% Confidence Interval); **Adj. HR:** Adjusted Hazard Ratio (95% CI)

**^a^ Mode of delivery**: number of events of the outcome of interest for each mode of delivery in parentheses

**^b^ Model 1:** Adjusted for maternal age, maternal origin, previous stillbirth, miscarriage or ectopic pregnancy, marital status, birth year and measures of socio-economic status including educational attainment, and mother and father’s gross income,

**^c^ Model 2:** Adjusted for Model 1 + medical complications in the first live birth including delivery type (singleton, twins or more), diabetes or gestational diabetes, placental abruption, placenta praevia and hypertensive disorders (including eclampsia and pre-eclampsia)

**^d^ Model 3:** Adjusted for Model 2 + gestational age and birth weight

***NOTE:** Where the number of events is less than 10 for maternally requested Cesarean, these were combined with the elective Cesarean group for analyses
