# Supplementary material for: Cesarean Section and Rate of Subsequent Stillbirth, Miscarriage, and Ectopic Pregnancy: A Danish Register-Based Cohort Study
Source: PLoS Med. 2014 Jul 1;11(7):e1001670. doi: 10.1371/journal.pmed.1001670 (PMC4077571; doi:10.1371/journal.pmed.1001670)
Supplement: Table S2 — Cesarean section and rate of subsequent miscarriage—additional analyses. (DOCX) [file pmed.1001670.s002.docx]

**Table S2:** Cesarean section and rate of subsequent miscarriage – additional analyses

| **^a^Mode of delivery** | **Cohort** Varies according to analyses | | | |
| --- | --- | --- | --- | --- |
| **Outcome: Miscarriage** | **Crude Model** | **Adj. HR (95% CI)** | | |
| Smoking adjustment (data from 1997-2010) (events n=31,411) | **Cr. HR (95% CI)** | **^b^ Model 1** | **^c^ Model 2** | **^d^ Model 3** |
| Spontaneous vaginal (n=20,897) | *ref* | *ref* | *ref* | *ref* |
| Operative vaginal (n=4,133) | 1.04 (1.01, 1.08) | 1.04 (1.01, 1.08) | 1.02 (0.99, 1.06) | 1.03 (1.00, 1.07) |
| Emergency Cesarean (n=4,615) | 0.95 (0.92, 0.98) | 0.95 (0.92, 0.99) | 0.99 (0.96, 1.02) | 1.00 (0.96, 1.03) |
| Elective Cesarean (n=1,636) | 0.88 (0.83, 0.92) | 0.88 (0.84, 0.93) | 0.96 (0.92, 1.01) | 0.96 (0.91, 1.01) |
| Maternally requested Cesarean (n=130) | 0.68 (0.57, 0.81) | 0.69 (0.58, 0.82) | 0.72 (0.60, 0.85) | 0.72 (0.61, 0.86) |
| BMI adjustment (data from 2003-2010) (events n=14,201) | **Cr. HR (95% CI)** | **Model 1** | **Model 2** | **Model 3** |
| Spontaneous vaginal (n=8,992) | *ref* | *ref* | *ref* | *ref* |
| Operative vaginal (n=1,960) | 1.08 (1.02, 1.13) | 1.06 (1.01, 1.11) | 1.04 (0.99, 1.10) | 1.05 (1.00, 1.10) |
| Emergency Cesarean (n=2,298) | 0.97 (0.93, 1.02) | 0.96 (0.92, 1.01) | 1.00 (0.95, 1.05) | 1.00 (0.96, 1.05) |
| Elective Cesarean (n=826) | 0.95 (0.88, 1.02) | 0.93 (0.86, 0.99) | 1.01 (0.94, 1.08) | 1.01 (0.94, 1.08) |
| Maternally requested Cesarean (n=125) | 0.68 (0.57, 0.81) | 0.68 (0.57, 0.81) | 0.71 (0.60, 0.85) | 0.71 (0.60, 0.85) |
| Fertility treatment adjustment (data from 1994-2005) (events n=34,603) | **Crude Model** | **Model 1** | **Model 2** | **Model 3** |
| Spontaneous vaginal (n=23,467) | *ref* | *ref* | *ref* | *ref* |
| Operative vaginal (n=4,759) | 1.03 (1.00, 1.06) | 1.03 (1.00, 1.07) | 1.02 (0.99, 1.05) | 1.03 (1.00, 1.06) |
| Emergency Cesarean (n=4,553) | 0.94 (0.91, 0.97) | 0.95 (0.92, 0.98) | 0.98 (0.95, 1.01) | 0.98 (0.95, 1.02) |
| Elective Cesarean (n=1,765) | 0.86 (0.82, 0.91) | 0.88 (0.84, 0.92) | 0.95 (0.90, 0.99) | 0.94 (0.89, 0.98) |
| Maternally requested Cesarean (n=59) | 0.66 (0.51, 0.86) | 0.67 (0.52, 0.86) | 0.70 (0.54, 0.90) | 0.70 (0.54, 0.90) |
| Restricted to smokers only (data from 1997-2010) (events n=4,979) | **Crude Model** | **Model 1** | **Model 2** | **Model 3** |
| Spontaneous vaginal (n=3,397) | *ref* | *ref* | *ref* | *ref* |
| Operative vaginal (n=607) | 1.03 (0.95, 1.13) | 1.08 (0.99, 1.17) | 1.07 (0.98, 1.16) | 1.08 (0.99, 1.18) |
| Emergency Cesarean (n=704) | 0.94 (0.87, 1.02) | 1.02 (0.94, 1.10) | 1.03 (0.95, 1.13) | 1.03 (0.95, 1.12) |
| Elective Cesarean (n=252) | 0.91 (0.80, 1.03) | 1.00 (0.88, 1.13) | 1.06 (0.94, 1.21) | 1.04 (0.92, 1.18) |
| Maternally requested Cesarean (n=19) | 0.84 (0.53, 1.31) | 0.95 (0.61, 1.50) | 0.99 (0.63, 1.56) | 0.98 (0.62, 1.55) |
| Restricted to maternal age >35 (data from 1982-2010) (events n=5,658) | **Crude Model** | **Model 1** | **Model 2** | **Model 3** |
| Spontaneous vaginal (n=3,332) | *ref* | *ref* | *ref* | *ref* |
| Operative vaginal (n=791) | 1.21 (1.12, 1.31) | 1.05 (0.97, 1.14) | 1.04 (0.96, 1.12) | 1.03 (0.95, 1.12) |
| Emergency Cesarean (n=1,052) | 0.97 (0.91, 1.04) | 0.92 (0.86, 0.99) | 0.97 (0.90, 1.04) | 0.97 (0.90, 1.04) |
| Elective Cesarean (n=440) | 0.80 (0.73, 0.88) | 0.76 (0.69, 0.83) | 0.83 (0.75, 0.91) | 0.83 (0.75, 0.92) |
| Maternally requested Cesarean (n=43) | 0.67 (0.49, 0.90) | 0.67 (0.49, 0.90) | 0.70 (0.52, 0.95) | 0.71 (0.52, 0.96) |

**Table S2:** Cesarean section and rate of subsequent miscarriage – additional analyses (continued)

| **^a^Mode of delivery** | **Cohort** Varies according to analyses | | | |
| --- | --- | --- | --- | --- |
| **Outcome: Miscarriage** | **Crude Model** | **Adj. HR (95% CI)** | | |
| Restricted to paternal age >45 (data from 1982-2010) (events n=1,349) | **Cr. HR (95% CI)** | **^b^ Model 1** | **^c^ Model 2** | **^d^ Model 3** |
| Spontaneous vaginal (n=889) | *ref* | *ref* | *ref* | *ref* |
| Operative vaginal (n=153) | 1.06 (0.89, 1.25) | 1.01 (0.85, 1.21) | 0.99 (0.83, 1.19) | 1.00 (0.84, 1.20) |
| Emergency Cesarean (n=223) | 0.93 (0.81, 1.08) | 0.93 (0.80, 1.08) | 0.95 (0.82, 1.10) | 0.94 (0.80, 1.09) |
| *****Elective Cesarean (n=84) | 0.68 (0.54, 0.85) | 0.70 (0.56, 0.88) | 0.73 (0.58, 0.92) | 0.72 (0.57, 0.91) |
| Restricted to BMI (<18.5) (data from 2003-2010) (events n=798) | **Cr. HR (95% CI)** | **Model 1** | **Model 2** | **Model 3** |
| Spontaneous vaginal (n=539) | *ref* | *ref* | *ref* | *ref* |
| Operative vaginal (n=113) | 1.08 (0.88, 1.32) | 1.07 (0.87, 1.32) | 1.05 (0.86, 1.29) | 1.06 (0.86, 1.30) |
| Emergency Cesarean (n=85) | 0.90 (0.72, 1.14) | 0.92 (0.73, 1.16) | 0.93 (0.73, 1.18) | 0.91 (0.72, 1.16) |
| *****Elective Cesarean (n=61) | 1.05 (0.81, 1.37) | 1.07 (0.82, 1.40) | 1.14 (0.87, 1.49) | 1.12 (0.86, 1.47) |
| Cohort effect (data restricted to 1982-1991) (events n=26,019) | **Crude Model** | **Model 1** | **Model 2** | **Model 3** |
| Spontaneous vaginal (n=21,547) | *ref* | *ref* | *ref* | *ref* |
| Operative vaginal (n=485) | 1.12 (1.02, 1.22) | 0.96 (0.87, 1.06) | 0.96 (0.87, 1.06) | 0.95 (0.87, 1.04) |
| Emergency Cesarean (n=2,971) | 0.96 (0.92, 1.00) | 0.99 (0.95, 1.03) | 1.01 (0.97, 1.05) | 0.99 (0.95, 1.03) |
| Elective Cesarean (n=1,016) | 0.91 (0.85, 0.97) | 0.96 (0.90, 1.03) | 0.99 (0.93, 1.06) | 0.99 (0.93, 1.05) |
| Cohort effect (data restricted to 1992-2001) (events n=30,536) | **Crude Model** | **Model 1** | **Model 2** | **Model 3** |
| Spontaneous vaginal (n=21,262) | *ref* | *ref* | *ref* | *ref* |
| Operative vaginal (n=4,292) | 0.99 (0.96, 1.03) | 1.01 (0.98, 1.05) | 1.01 (0.97, 1.04) | 1.01 (0.98, 1.05) |
| Emergency Cesarean (n=3,530) | 0.91 (0.88, 0.94) | 0.94 (0.90, 0.97) | 0.97 (0.94, 1.01) | 0.97 (0.94, 1.01) |
| Elective Cesarean (n=1,452) | 0.83 (0.78, 0.87) | 0.86 (0.82, 0.91) | 0.93 (0.88, 0.99) | 0.92 (0.87, 0.98) |
| Cohort effect (data restricted to 2002-2010) (events n=16,851) | **Crude Model** | **Model 1** | **Model 2** | **Model 3** |
| Spontaneous vaginal (n=10,731) | *ref* | *ref* | *ref* | *ref* |
| Operative vaginal (n=2,328) | 1.07 (1.03, 1.12) | 1.06 (1.01, 1.11) | 1.04 (1.00, 1.09) | 1.05 (1.00, 1.10) |
| Emergency Cesarean (n=2,677) | 0.96 (0.92, 1.00) | 0.95 (0.91, 0.99) | 0.98 (0.94, 1.02) | 0.99 (0.95, 1.03) |
| Elective Cesarean (n=985) | 0.93 (0.87, 0.99) | 0.92 (0.86, 0.98) | 1.00 (0.94, 1.07) | 1.00 (0.93, 1.07) |
| Maternally requested Cesarean (n=130) | 0.68 (0.58, 0.81) | 0.69 (0.58, 0.82) | 0.72 (0.60, 0.85) | 0.72 (0.61, 0.86) |

**Table S2:** Cesarean section and rate of subsequent miscarriage – additional analyses (continued)

| Miscarriage definition (data restricted to 2004-2010) (events n=11,580) | **Crude Model** | **Model 1** | **Model 2** | **Model 3** |
| --- | --- | --- | --- | --- |
| Spontaneous vaginal (n=7,310) | *ref* | *ref* | *ref* | *ref* |
| Operative vaginal (n=1,593) | 1.07 (1.02, 1.13) | 1.05 (1.00, 1.11) | 1.04 (0.98, 1.09) | 1.04 (0.99, 1.10) |
| Emergency Cesarean (n=1,900) | 0.98 (0.94, 1.04) | 0.97 (0.93, 1.03) | 1.01 (0.96, 1.06) | 1.01 (0.96, 1.07) |
| Elective Cesarean (n=668) | 0.96 (0.88, 1.04) | 0.93 (0.86, 1.01) | 1.01 (0.93, 1.10) | 1.01 (0.94, 1.10) |
| Maternally requested Cesarean (n=109) | 0.66 (0.55, 0.80) | 0.66 (0.55, 0.80) | 0.69 (0.57, 0.83) | 0.69 (0.57, 0.83) |

**Data refer to: Cr. HR:** Crude Hazard Ratio (95% Confidence Interval); **Adj. HR:** Adjusted Hazard Ratio (95% CI)

**^a^ Mode of delivery**: number of events of the outcome of interest for each mode of delivery in parentheses

**^b^ Model 1:** Adjusted for maternal age, maternal origin, previous stillbirth, miscarriage or ectopic pregnancy, marital status, birth year^,^ and measures of socio-economic status including educational attainment, and mother and father’s gross income,

**^c^ Model 2:** Adjusted for Model 1 + medical complications in the first live birth including delivery type (singleton, twins or more), diabetes or gestational diabetes, placental abruption, placenta praevia and hypertensive disorders (including eclampsia and pre-eclampsia)

**^d^ Model 3:** Adjusted for Model 2 + gestational age and birth weight

***NOTE:** Where the number of events is less than 10 for maternally requested Cesarean, these were combined with the elective Cesarean group for analyses
