# Supplementary material for: Cesarean Section and Rate of Subsequent Stillbirth, Miscarriage, and Ectopic Pregnancy: A Danish Register-Based Cohort Study
Source: PLoS Med. 2014 Jul 1;11(7):e1001670. doi: 10.1371/journal.pmed.1001670 (PMC4077571; doi:10.1371/journal.pmed.1001670)
Supplement: Table S3 — Cesarean section and rate of subsequent ectopic pregnancy—additional analyses. (DOCX) [file pmed.1001670.s003.docx]

**Table S3:** Cesarean section and rate of subsequent ectopic pregnancy – additional analyses

| **^a^Mode of delivery** | **Cohort** Varies according to analyses | | | |
| --- | --- | --- | --- | --- |
| **Outcome: Ectopic pregnancy** | **Crude Model** | **Adj. HR (95% CI)** | | |
| Smoking adjustment (data from 1997-2010) (events n=4,596) | **Cr. HR (95% CI)** | **^b^ Model 1** | **^c^ Model 2** | **^d^ Model 3** |
| Spontaneous vaginal (n=2,967) | *ref* | *ref* | *ref* | *ref* |
| Operative vaginal (n=560) | 1.00 (0.92, 1.10) | 1.03 (0.94, 1.13) | 1.02 (0.93, 1.12) | 1.05 (0.96, 1.15) |
| Emergency Cesarean (n=764) | 1.10 (1.02, 1.19) | 1.12 (1.03, 1.21) | 1.14 (1.05, 1.24) | 1.17 (1.07, 1.27) |
| Elective Cesarean (n=281) | 1.06 (0.94, 1.20) | 1.09 (0.97, 1.23) | 1.13 (1.00, 1.28) | 1.13 (1.00, 1.27) |
| Maternally requested Cesarean (n=24) | 0.90 (0.60, 1.34) | 0.97 (0.65, 1.46) | 1.02 (0.68, 1.52) | 1.02 (0.68, 1.53) |
| BMI adjustment (data from 2003-2010) (events n=2,081) | **Cr. HR (95% CI)** | **Model 1** | **Model 2** | **Model 3** |
| Spontaneous vaginal (n=1,296) | *ref* | *ref* | *ref* | *ref* |
| Operative vaginal (n=253) | 1.00 (0.67, 1.51) | 1.00 (0.87, 1.14) | 0.99 (0.86, 1.13) | 1.02 (0.89, 1.16) |
| Emergency Cesarean (n=366) | 1.06 (0.94, 1.19) | 1.09 (0.97, 1.23) | 1.13 (1.00, 1.27) | 1.15 (1.02, 1.31) |
| Elective Cesarean (n=142) | 1.11 (0.93, 1.32) | 1.14 (0.96, 1.36) | 1.20 (1.00, 1.43) | 1.20 (1.00, 1.43) |
| Maternally requested Cesarean (n=24) | 0.89 (0.60, 1.34) | 1.00 (0.67, 1.51) | 1.05 (0.70, 1.58) | 1.07 (0.71, 1.60) |
| Fertility treatment adjustment (data from 1994-2005) (events n=5,019) | **Crude Model** | **Model 1** | **Model 2** | **Model 3** |
| Spontaneous vaginal (n=3,245) | *ref* | *ref* | *ref* | *ref* |
| Operative vaginal (n=659) | 1.02 (0.93, 1.10) | 1.08 (0.99, 1.17) | 1.06 (0.98, 1.16) | 1.08 (0.99, 1.17) |
| Emergency Cesarean (n=766) | 1.04 (0.96, 1.13) | 1.08 (1.00, 1.17) | 1.13 (1.04, 1.22) | 1.15 (1.06, 1.25) |
| *****Elective Cesarean (n=349) | 1.03 (0.92, 1.15) | 1.11 (0.99, 1.24) | 1.20 (1.07, 1.35) | 1.19 (1.06, 1.34) |
| Restricted to smokers only (data from 1997-2010) (events n=1,033) | **Crude Model** | **Model 1** | **Model 2** | **Model 3** |
| Spontaneous vaginal (n=691) | *ref* | *ref* | *ref* | *ref* |
| Operative vaginal (n=113) | 0.95 (0.78, 1.15) | 0.98 (0.80, 1.19) | 0.98 (0.80, 1.20) | 1.00 (0.81, 1.22) |
| Emergency Cesarean (n=175) | 1.15 (0.98, 1.36) | 1.21 (1.03, 1.44) | 1.22 (1.03, 1.45) | 1.26 (1.05, 1.50) |
| *****Elective Cesarean (n=54) | 0.87 (0.66, 1.16) | 0.95 (0.71, 1.26) | 0.96 (0.72, 1.28) | 0.95 (0.72, 1.27) |
| Restricted to maternal age >35 (data from 1982-2010) (events n=619) | **Crude Model** | **Model 1** | **Model 2** | **Model 3** |
| Spontaneous vaginal (n=362) | *ref* | *ref* | *ref* | *ref* |
| Operative vaginal (n=80) | 1.12 (0.88, 1.43) | 0.97 (0.76, 1.24) | 0.95 (0.74, 1.22) | 0.94 (0.73, 1.20) |
| Emergency Cesarean (n=119) | 1.02 (0.83, 1.26) | 0.93 (0.75, 1.14) | 0.93 (0.75, 1.15) | 0.94 (0.76, 1.17) |
| *****Elective Cesarean (n=58) | 0.88 (0.66, 1.16) | 0.75 (0.57, 1.00) | 0.76 (0.57, 1.02) | 0.79 (0.59, 1.05) |

**Table S3:** Cesarean section and rate of subsequent ectopic pregnancy – additional analyses (continued)

| **^a^Mode of delivery** | **Cohort^e^** Varies according to analyses | | | |
| --- | --- | --- | --- | --- |
| **Outcome: Ectopic pregnancy** | **Crude Model** | **Adj. HR (95% CI)** | | |
| Cohort effect (data restricted to 1982-1991) (events n=4,950) | **Cr. HR (95% CI)** | **^b^ Model 1** | **^c^ Model 2** | **^d^ Model 3** |
| Spontaneous vaginal (n=4,090) | *ref* | *ref* | *ref* | *ref* |
| Operative vaginal (n=84) | 1.01 (0.82, 1.26) | 0.90 (0.72, 1.14) | 0.90 (0.71, 1.14) | 0.92 (0.74, 1.15) |
| Emergency Cesarean (n=574) | 0.95 (0.87, 1.04) | 1.00 (0.92, 1.09) | 1.01 (0.93, 1.10) | 1.03 (0.94, 1.13) |
| Elective Cesarean (n=202) | 0.93 (0.80, 1.07) | 0.96 (0.84, 1.11) | 0.99 (0.85, 1.14) | 1.00 (0.87, 1.16) |
| Cohort effect (data restricted to 1992-2001) (events n=4,467) | **Crude Model** | **Model 1** | **Model 2** | **Model 3** |
| Spontaneous vaginal (n=2,987) | *ref* | *ref* | *ref* | *ref* |
| Operative vaginal (n=614) | 1.01 (0.92, 1.10) | 1.05 (0.96, 1.15) | 1.05 (0.96, 1.15) | 1.07 (0.98, 1.16) |
| Emergency Cesarean (n=588) | 1.06 (0.97, 1.15) | 1.07 (0.97, 1.16) | 1.07 (0.98, 1.17) | 1.09 (0.99, 1.19) |
| Elective Cesarean (n=278) | 1.09 (0.97, 1.24) | 1.14 (1.01, 1.29) | 1.16 (1.03, 1.32) | 1.15 (1.01, 1.31) |
| Cohort effect (data restricted to 2002-2010) (events n=2,460) | **Crude Model** | **Model 1** | **Model 2** | **Model 3** |
| Spontaneous vaginal (n=1,522) | *ref* | *ref* | *ref* | *ref* |
| Operative vaginal (n=298) | 0.97 (0.85, 1.09) | 1.00 (0.88, 1.13) | 0.99 (0.88, 1.12) | 1.02 (0.90, 1.16) |
| Emergency Cesarean (n=443) | 1.11 (1.00, 1.23) | 1.12 (1.01, 1.25) | 1.16 (1.04, 1.29) | 1.19 (1.07, 1.33) |
| Elective Cesarean (n=173) | 1.14 (0.97, 1.33) | 1.18 (1.00, 1.38) | 1.24 (1.05, 1.45) | 1.22 (1.04, 1.44) |
| Maternally requested Cesarean (n=24) | 0.89 (0.60, 1.33) | 0.97 (0.65, 1.46) | 1.02 (0.68, 1.53) | 1.02 (0.68, 1.54) |

**Data refer to: Cr. HR:** Crude Hazard Ratio (95% Confidence Interval); **Adj. HR:** Adjusted Hazard Ratio (95% CI)

**^a^ Mode of delivery**: number of events of the outcome of interest for each mode of delivery in parentheses

**^b^ Model 1:** Adjusted for maternal age, maternal origin, previous stillbirth, miscarriage or ectopic pregnancy, marital status, birth year and measures of socio-economic status including educational attainment, and mother and father’s gross income

**^c^ Model 2:** Adjusted for Model 1 + medical complications in the first live birth including delivery type (singleton, twins or more), diabetes or gestational diabetes, placental abruption, placenta praevia and hypertensive disorders (including eclampsia and pre-eclampsia)

**^d^ Model 3:** Adjusted for Model 2 + gestational age and birth weight

***NOTE:** Where the number of events is less than 10 for maternally requested Cesarean, these were combined with the elective Cesarean group for analyses
