# Supplementary material for: Cesarean Section and Rate of Subsequent Stillbirth, Miscarriage, and Ectopic Pregnancy: A Danish Register-Based Cohort Study
Source: PLoS Med. 2014 Jul 1;11(7):e1001670. doi: 10.1371/journal.pmed.1001670 (PMC4077571; doi:10.1371/journal.pmed.1001670)
Supplement: Table S4 — Cesarean section and rate of subsequent stillbirth, miscarriage, or ectopic pregnancy—sensitivity analyses excluding women with a history of pregnancy loss. (DOCX) [file pmed.1001670.s004.docx]

**Table S4** Cesarean section and rate of subsequent stillbirth, miscarriage or ectopic pregnancy – sensitivity analysis excluding women with a history of pregnancy loss **^e^**

| **^a^Mode of delivery** | **Cohort:** 1982-2010 (n=812,318 women) | | | |
| --- | --- | --- | --- | --- |
| **Outcome** | **Crude Model** | **Adj. HR (95% CI)** | | |
| **Stillbirth** (n=1,943 events) | **Cr. HR (95% CI)** | **^b^ Model 1** | **^c^ Model 2** | **^d^ Model 3** |
| Spontaneous vaginal (n=1,365) | *ref* | *ref* | *ref* | *ref* |
| Operative vaginal (n=169) | 1.04 (0.89, 1.22) | 1.07 (0.90, 1.26) | 1.04 (0.88, 1.22) | 1.05 (0.89, 1.24) |
| All Cesarean sections (n=409) | 1.17 (1.05, 1.31) | 1.25 (1.12, 1.40) | 1.21 (1.08, 1.36) | 1.15 (1.02, 1.29) |
| Emergency Cesarean (n=298) | 1.20 (1.06, 1.36) | 1.27 (1.12, 1.44) | 1.23 (1.08, 1.40) | 1.17 (1.02, 1.33) |
| *****Elective Cesarean (n=111) | 1.10 (0.90, 1.33) | 1.22 (1.00, 1.48) | 1.17 (0.96, 1.43) | 1.10 (0.90, 1.34) |
| **Miscarriage** (n=70,873 events) | **Crude Model** | **Model 1** | **Model 2** | **Model 3** |
| Spontaneous vaginal (n=51,835) | *ref* | *ref* | *ref* | *ref* |
| Operative vaginal (n=6,893) | 1.10 (1.07, 1.13) | 1.04 (1.01, 1.06) | 1.03 (1.00, 1.05) | 1.03 (1.00, 1.06) |
| All Cesarean sections (n=12,145) | 0.94 (0.92, 0.96) | 0.95 (0.93, 0.96) | 0.98 (0.96, 1.00) | 0.98 (0.96, 1.00) |
| Emergency Cesarean (n=8,784) | 0.95 (0.93, 0.98) | 0.96 (0.94, 0.98) | 0.99 (0.97, 1.01) | 0.99 (0.96, 1.01) |
| Elective Cesarean (n=3,244) | 0.89 (0.86, 0.92) | 0.91 (0.87, 0.94) | 0.97 (0.93, 1.00) | 0.96 (0.92, 0.99) |
| Maternally requested Cesarean (n=117) | 0.66 (0.55, 0.79) | 0.67 (0.55, 0.80) | 0.69 (0.58, 0.83) | 0.69 (0.58, 0.83) |
| **Ectopic pregnancy** (n=9,688 events) | **Crude Model** | **Model 1** | **Model 2** | **Model 3** |
| Spontaneous vaginal (n=7,098) | *ref* | *ref* | *ref* | *ref* |
| Operative vaginal (n=793) | 0.94 (0.87, 1.01) | 1.04 (0.96, 1.12) | 1.03 (0.96, 1.12) | 1.04 (0.97, 1.13) |
| All Cesarean sections (n=1,797) | 0.99 (0.94, 1.05) | 1.11 (1.05, 1.17) | 1.14 (1.08, 1.20) | 1.15 (1.09, 1.21) |
| Emergency Cesarean (n=1,275) | 0.99 (0.94, 1.06) | 1.09 (1.02, 1.15) | 1.11 (1.05, 1.18) | 1.13 (1.06, 1.20) |
| *Elective Cesarean (n=522) | 0.99 (0.91, 1.09) | 1.16 (1.06, 1.26) | 1.21 (1.10, 1.32) | 1.20 (1.10, 1.32) |

**Data refer to: Cr. HR:** Crude Hazard Ratio (95% Confidence Interval); **Adj. HR:** Adjusted Hazard Ratio (95% CI)

**^a^ Mode of delivery**: number of events of the outcome of interest for each mode of delivery in parentheses

**^b^ Model 1:** Adjusted for maternal age, maternal origin, marital status, birth year and measures of socio-economic status including educational attainment, and mother and father’s gross income

**^c^ Model 2:** Adjusted for Model 1 + medical complications in the first live birth including delivery type (singleton, twins or more), diabetes or gestational diabetes, placental abruption, placenta praevia and hypertensive disorders (including eclampsia and pre-eclampsia)

**^d^ Model 3:** Adjusted for Model 2 + gestational age and birth weight

^e^ **History of pregnancy loss:** women with a prior stillbirth, miscarriage or ectopic pregnancy before the index live birth were excluded (n=20,678)

***NOTE:** Where the number of events is less than 10 for maternally requested Cesarean, these were combined with the elective Cesarean group for analyses
