# Supplementary material for: Cesarean Section and Rate of Subsequent Stillbirth, Miscarriage, and Ectopic Pregnancy: A Danish Register-Based Cohort Study
Source: PLoS Med. 2014 Jul 1;11(7):e1001670. doi: 10.1371/journal.pmed.1001670 (PMC4077571; doi:10.1371/journal.pmed.1001670)
Supplement: Text S1 — Cesarean section and rate of subsequent stillbirth, miscarriage, or ectopic pregnancy—additional analyses. (DOCX) [file pmed.1001670.s005.docx]

**Supporting Information**

Supplement to: O’Neill SM, Agerbo E, Kenny LC, Henriksen TB, Kearney PM, Greene RA, Mortensen PB, and Khashan AS. Cesarean Section and Rate of Subsequent Stillbirth, Miscarriage and Ectopic Pregnancy: a Danish Register-based Cohort Study

**Cesarean section and rate of subsequent stillbirth – additional adjusted and exploratory analyses**

**Smoking adjustment**

When smoking was added to the models (Table S1) an increased rate of stillbirth was found in emergency Cesarean section (HR 1.07, 95% CI 0.87, 1.31) and elective Cesarean section (HR 1.11, 95% CI 0.82, 1.49). These were both however, statistically nonsignificant.

**BMI adjustment**

Adjustment by BMI (Table S1) found an increased rate of stillbirth in elective Cesarean only (HR 1.05, 95% CI 0.69, 1.60) compared to prior SVD.

**Previous fertility treatment adjustment**

Previous fertility treatment (Table S1) resulted in an increased rate of stillbirth among emergency (HR 1.16, 95% CI 0.96, 1.41) and elective Cesarean section (HR 1.15, 95% CI 0.87, 1.52), although both were below the level of statistical significance.

**Subgroup analyses - smokers only**

When the cohort was restricted only to include women who were smokers (Table S1) an increased rate of stillbirth was found in the prior elective Cesarean section group (HR 1.64, 95% CI 0.93, 2.90).

**Subgroup analyses - term deliveries only**

When only term deliveries (>37 weeks <41 weeks gestation) were analysed (Table S1), a significantly increased rate of stillbirth was found among emergency Cesarean section only (HR 1.26, 95% CI 1.06, 1.50).

**Subgroup analyses – preterm deliveries only**

When only preterm deliveries (<37 weeks gestation) were analysed (Table S1), a significantly increased rate of stillbirth was found among elective Cesarean section only (HR 1.90, 95% CI 1.22, 2.98). An increased rate of stillbirth in prior emergency Cesarean (HR 1.37, 95% CI 0.94, 1.99) and prior operative vaginal delivery (HR 1.65, 95% CI 0.78, 3.48) was also found however, neither reached statistical significance.

**Subgroup analyses – post term deliveries only**

An increased but insignificant rate of stillbirth was found in prior elective Cesarean only (HR 1.11, 95% CI 0.53, 2.36) (Table S1).

**Subgroup analyses – cohort divided in to three time periods (1982-1991), (1992-2001), (2002-2010)**

In the earliest (1982-1991) time period, prior emergency Cesarean section (HR 1.19, 95% CI 0.97, 1.47) only was associated with an increased rate of stillbirth. This was true also for the middle (1992-2001) time period for emergency Cesarean section only (HR 1.13, 95% CI 0.92, 1.40). In the later years (2002-2010) however, the increased rate shifted to prior elective Cesarean section (HR 1.29, 95% CI 0.90, 1.85) only (Table S1).

**Subgroup analyses – definition of stillbirth changed from 28 to 22 weeks gestation (data restricted from 2004 to 2010)**

A change in definition from 2004 onwards yielded an increased but insignificant rate of stillbirth (Table S1) among elective Cesarean section only (HR 1.13, 95% CI 0.71, 1.77).

**Cesarean section and rate of subsequent miscarriage – additional adjusted and exploratory analyses**

**Smoking adjustment**

When smoking was added to the crude and adjusted models, no significantly increased rate of miscarriage was found across all modes of delivery (Table S2), with maternally requested Cesarean section having a decreased rate of subsequent miscarriage (HR 0.72, 95% CI 0.61, 0.86).

**BMI adjustment**

When BMI was added to the analyses, there was no increased rate of miscarriage found across all modes of delivery (Table S2), with maternally requested Cesarean section showing a decreased rate of subsequent miscarriage (HR 0.71, 95% CI 0.60, 0.85).

**Previous fertility treatment adjustment**

When access to prior fertility treatment before the first live birth was adjusted for in the analyses, no significantly increased rate of miscarriage was found across all modes of delivery. Maternally requested Cesarean section was associated with a reduced rate of subsequent miscarriage (HR 0.70, 95% CI 0.54, 0.90).

**Subgroup analyses - smokers only**

When the cohort was restricted to include smokers only, no significantly increased rate of subsequent miscarriage was reported across all modes of delivery (Table S2).

**Subgroup analyses –advanced maternal age only (35+ years)**

When the cohort was restricted to include only women of advanced maternal age (Table S2) no increased rate of subsequent miscarriage was found, with a prior elective (HR 0.83, 95% CI 0.75, 0.92) and maternally requested Cesarean (HR 0.71, 95% CI 0.52, 0.96) having decreased rates.

**Subgroup analyses –advanced paternal age only (45+ years)**

Where the analyses was restricted to include women whose partner or spouse was of advanced paternal age no significantly increased rate of miscarriage was found across all modes of delivery, with elective Cesarean section (HR 0.72, 95% CI 0.57, 0.91) associated with a reduced rate of miscarriage (Table S2).

**Subgroup analyses –underweight BMI only (<18.5)**

Where women with an underweight BMI only were included, no significantly increased rate of miscarriage across all modes of delivery was found (Table S2).

**Subgroup analyses – Cohort divided in to three time periods (1982-1991), (1992-2001), (2002-2010)**

No significantly increased rate of miscarriage was found across all three time periods.

**Subgroup analyses – Definition of miscarriage changed from 28 weeks to 22 weeks gestation (data restricted from 2004 to 2010)**

The findings overall did not change in the subgroup analyses testing the effect of the change in miscarriage definition in 2004 (Table S2).

**Cesarean section and rate of subsequent ectopic pregnancy – additional adjusted and exploratory analyses**

**Smoking adjustment**

When smoking was adjusted for in the analyses, a significantly increased rate of ectopic pregnancy was found among prior emergency Cesarean (HR 1.17, 95% CI 1.07, 1.27) only (Table S3).

**BMI adjustment**

When BMI was considered in the analyses, an increased rate of subsequent ectopic pregnancy was found in emergency Cesarean (HR 1.15, 95% CI 1.02, 1.31) and prior elective Cesarean (HR 1.20, 95% CI 1.00, 1.43) only (Table S3).

**Previous fertility treatment adjustment**

History of access to fertility treatment before the first live birth was associated with an increased rate of subsequent ectopic pregnancy in women with prior emergency (HR 1.15, 95% CI 1.06, 1.25) and prior elective Cesarean (HR 1.19, 95% CI 1.06, 1.34) only (Table S3).

**Subgroup analyses - smokers only**

When the analyses was restricted to include smokers only (Table S3) a significantly increased rate of ectopic pregnancy was found among prior emergency Cesarean only (HR 1.26, 95 %CI 1.05, 1.50).

**Subgroup analyses –advanced maternal age only (35+ years)**

No increased rate of subsequent ectopic pregnancy was found for all modes of delivery when the cohort was restricted to women of advanced maternal age (Table S3).

**Subgroup analyses – cohort divided in to three time periods (1982-1991), (1992-2001), (2002-2010)**

In the earliest time period (Table S3) no significantly increased rate of ectopic pregnancy was found. From 1992-2001 and 2002-2010, a statistically significant increased rate of ectopic pregnancy was found in emergency and elective Cesarean section.

**ALL OUTCOMES**

**Sensitivity analyses – excluding women with a history of pregnancy loss**

When women with a history of stillbirth, miscarriage or ectopic pregnancy before the first live birth were excluded, no change in the overall results was found (Table S4).
